# Morphological Evolution of Metal‐Organic Frameworks into Hedrite, Sheaf and Spherulite Superstructures with Localized Different Coloration

**DOI:** 10.1002/chem.202403577

**Published:** 2024-12-13

**Authors:** Naveen Malik, Linda J. W. Shimon, Lothar Houben, Anna Kossoy, Iddo Pinkas, Ifat Kaplan‐Ashiri, Tatyana Bendikov, Michal Lahav, Milko E. van der Boom

**Affiliations:** ^1^ Department of Molecular Chemistry and Materials Science Weizmann Institute of Science Rehovot 7610001 Israel; ^2^ Department of Chemistry College of Engineering and Technology SRM Institute of Science and Technology Kattankulathur 603203 India; ^3^ Department of Chemical Research Support Weizmann Institute of Science Rehovot 7610001 Israel

**Keywords:** Hyperbranched morphologies, Secondary crystallization, Crystal splitting, Superstructures

## Abstract

The branched metal‐organic frameworks (MOFs) are the first superstructures of this kind, and the growth mechanism may explain crystal shapes of other materials. The mechanism of the formation of fascinating structures having a hedrite, sheaf or spherulite appearance are detailed. The branching can be controlled, resulting in crystals that either exhibit multiple generations of branching or a single generation. These structures might result from an increasing number of defects on fast‐grown rods. As the basal facets become less reactive, material is added to the prism facets, leading to secondary nucleation and triangular branches. These triangular structures are connected to the rod surface, growing longer than the central rod. Electron diffraction analyses show that the sheafs are polycrystalline structures with their fantails consisting of single‐crystalline nanorods deviating gradually from each other in their orientation. The crystallographic structure consists of channels with opposite handedness. The accessibility of the nanochannels and the porosity of the superstructures are demonstrated by chromophore diffusion into the channels. The confinement and alignment of the chromophores inside the channels resulted in polarized‐light dependent coloration of the crystals; the polycrystallinity generated areas having different optical properties.

## Graphical abstract

The hyperbranched metal‐organic superstructures reported here exhibit diverse morphologies, including hedrites, sheafs, and spherulites. These structures are formed via non‐crystallographic branching, which represents a distinct growth mechanism typically observed in many materials and is being reported for the first time in metal‐organic frameworks.

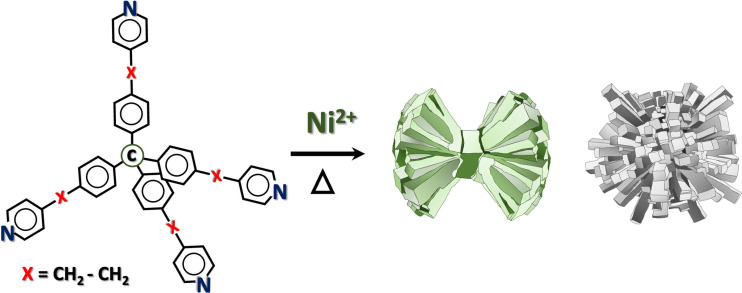

## Introduction

Many different types of materials form sheaf‐like and spherulite superstructures.^[1]^ Examples of hyperbranched crystalline natural materials are minerals including stilbite, calcium oxalate, uric acid, fluorapatite.[^2^, ^3^] Such morphologies have also been observed in inorganic salts: Bi_2_S_3_,^[4]^ metal phosphides,[^5^, ^6^] BaSO_4_,^[7]^ and LaVO_4_,^[8]^ just to name of few. Polymers including proteins and metals are known to appear also as spherulites.[^9^, ^10^, ^11^] The properties of these crystalline materials have been shown to be strongly dependent on their morphologies. For example, mesoporous sheaf‐like cobalt oxide functions as superior anode for rechargeable lithium‐ion batteries.^[12]^ Laeri *et al* showed that dye‐loaded zeolite AlPO4‐5 can function as microlaser, where only individual zeolite crystals with a dumbbell‐like shape show laser emission.^[13]^

The evolution of complex morphologies requires control over the synthetic parameters such as solvents, temperature and additives. Different mechanisms have been proposed for the formation of sheaf‐like and spherulites superstructures, including (*i*) crystal splitting and (*ii*) non‐crystallographic branching.[^1^, ^14^, ^15^, ^16^] Anisotropic sheaf‐like structures are intermediates in the formation of spherulites. The former mechanism (*i*) results in splitting of a parent crystal (=simple splitting) and the structures develop into rods or lamellas at the tips forming hedrites, sheafs, and spherulites. The latter process (*ii*) termed non‐crystallographic branching was postulated by Keith and Padden and is believed to involve secondary nucleation of crystals on a seed with a slight misorientation.^[16]^

We found only a few literature reports on metal‐organic frameworks (MOFs) with sheaf‐like and spherulite shapes. You, Han and others reported such shaped MOFs and coordination polymers based on lanthanides as metal ion centers and benzenetricarboxylate (1,3,5‐BTC) as coordinating ligand.[^17^, ^18^, ^19^, ^20^] These materials exhibit tunable light emission. Another recent example is the spherulite MOF‐74 reported by Zhou *et al* formed via small‐angle branching and radial growth.^[21]^ The spherulite MOF‐74 has a fused core and surface fibers. Under polarized light, these crystals exhibit a solvent‐dependent Maltese cross pattern as is common for spherulites. The paucity of examples of sheaf and spherulite shaped MOFs is surprising. Coordination chemistry has been extensively used for the formation of numerous MOFs during the past decades.[^22^, ^23^, ^24^] These crystalline structures are explored for energy storage, carbon capture, catalysis, etc. MOFs are also promising materials for optical applications. Adsorption of dyes into their voids resulted in nonlinear optical and fluorescence properties, and Förster resonance energy transfer (FRET). The alignment of dyes in nanosized channels resulted in optical anisotropy.^[25]^ Their properties can be tuned by using different metal centers and organic linkers. Although great progress has been made in the control of packing, network structures, and porosity; the control over the morphology and uniformity of such useful crystals remains an intriguing challenge. The formation of new hyperbranched MOFs can lead to new functionalities as shown in this study. Our group has recently shown that the coordination chemistry of tetrahedral pyridine‐based ligands with first row, bivalent metals (i. e. nickel and copper) resulted in uniform structures.[^25^, ^26^, ^27^, ^28^] These studies include the preparation of rare single crystals having multidomain morphologies. Until now, we have not observed branched superstructures with our pyridine‐based ligands.

**Scheme 1:**
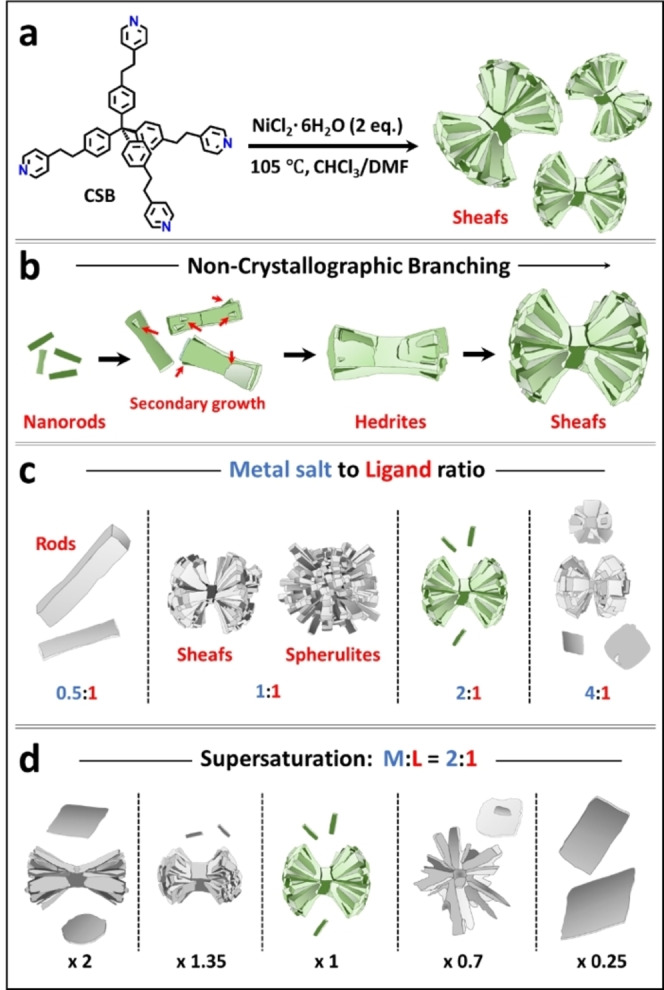
(a) Solvothermal synthesis of sheaf‐like metal‐organic crystals. (b) Non‐crystallographic branching. The arrows indicate the initial appearance of fantail structures. (c, d) The crystal morphology and dimensions are related to the ligand‐to‐metal salt ratio and the concentration of the ligand. The crystals shown in green were obtained using a metal salt‐to‐ligand molecular ratio of 2 : 1 with CSB=0.6 mM. For (d), the numbers below the drawings refer to the concentrations of the components used to grow the sheafs.

We introduce a full set of hyperbranched metal‐organic superstructures. The hedrites, sheafs and spherulites shown in Scheme 1 are formed from rods that evolve into thermodynamically favored branched morphologies. The size and morphology of these crystals are manipulated by using the same building blocks under different reaction conditions. The hyperbranched structures were obtained without the use of external modulators. The crystallinity of our materials is evident from detailed crystallographic and electron diffraction studies of sections of the crystals. These studies show that the crystallographic orientation of the nanorods of the fantails deviates slightly from each other. However, their crystallographic structure is identical. These structural features are consistent with non‐crystallographic branching, except such MOFs are unusual. The morphology can be controlled: crystals were formed with several generations of branching or with as single generation. The lack of subsequent branching of branches is the most characteristic feature of a spherulite. We postulate that their growth differs from the accepted mechanism for hyperbranched crystalline minerals where actual splitting of these materials occurs.[^1^, ^2^, ^3^] It is likely that defects formed during the fast growth of initial MOFs act as secondary nucleation centers.

We took advantage of the polycrystallinity and the porosity of the sheaf‐like superstructure to demonstrate areas with different optical activity under polarized light. These functionalized crystals were obtained by diffusion of a chromophore (=sodium resorufin, **SR**) into nanosized channels resulted in the alignment of these dyes as constrained by the crystallographic structure. As the branches are not parallel to each other and orientated differently from the central rod there is no global polarization alignment. These structural features are reflected in the optical properties of the sub‐units of crystals.

## Results and Discussion

**Figure 1:**
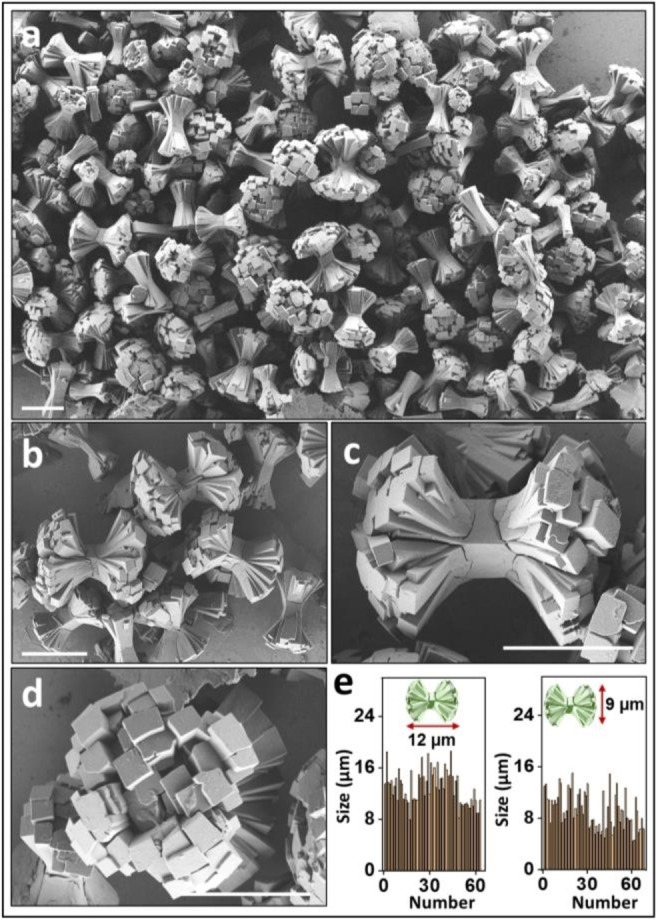
(a–d) Scanning electron microscopy (SEM) images of sheaf‐like **MOF‐NiCl_2_**. Scale bar=10 *μ*m. (e) Histograms of the length and width distribution. The crystals were obtained using a metal salt‐to‐ligand molecular ratio of 2 : 1, with **CSB**=0.6 mM, at 105 °C for 5 days.

The sheaf‐like metallo‐organic crystals (**MOF‐NiCl_2_**) are formed under solvothermal reaction conditions. The ligand (**CSB**)^[29]^ was reacted with nickel dichloride (NiCl_2_) in a chloroform/dimethyl‐formamide (DMF) solution (1 : 3 v/v) for 5 days at 105 °C. The molar ratio of NiCl_2_:**CSB=**2 : 1 (Scheme 1a). Scanning electron microscopy (SEM) and optical images showed the formation of sheaf‐like superstructures with two fantails consisting of a closely packed bundle of nanorods (Figures 1a–d, S1, S2). These nanorod aggregates are arranged around one central rod. Analysis of the morphology indicates a high level of uniformity: the sheafs have a length of 12.0±2.5 *μ*m and the width of the fantails is in the range of 9.2±2.8 *μ*m (Figure 1e). Each fantail consists of 25±4 nanorods, as indicated by examining 20 sheafs. The nanorods of the fantails have cross sections 1.2±0.3 *μ*m and the length of the free bridge of the central rod is 4.6±0.9 *μ*m (estimated from 30 crystals). Moreover, we see that both fantails of the individual sheaf‐like superstructures are similar in size and composition. We also observed some relatively small, isolated rods (*l*=4.0±1.1 *μ*m) as a minor product (Figure S2).

**Figure 2:**
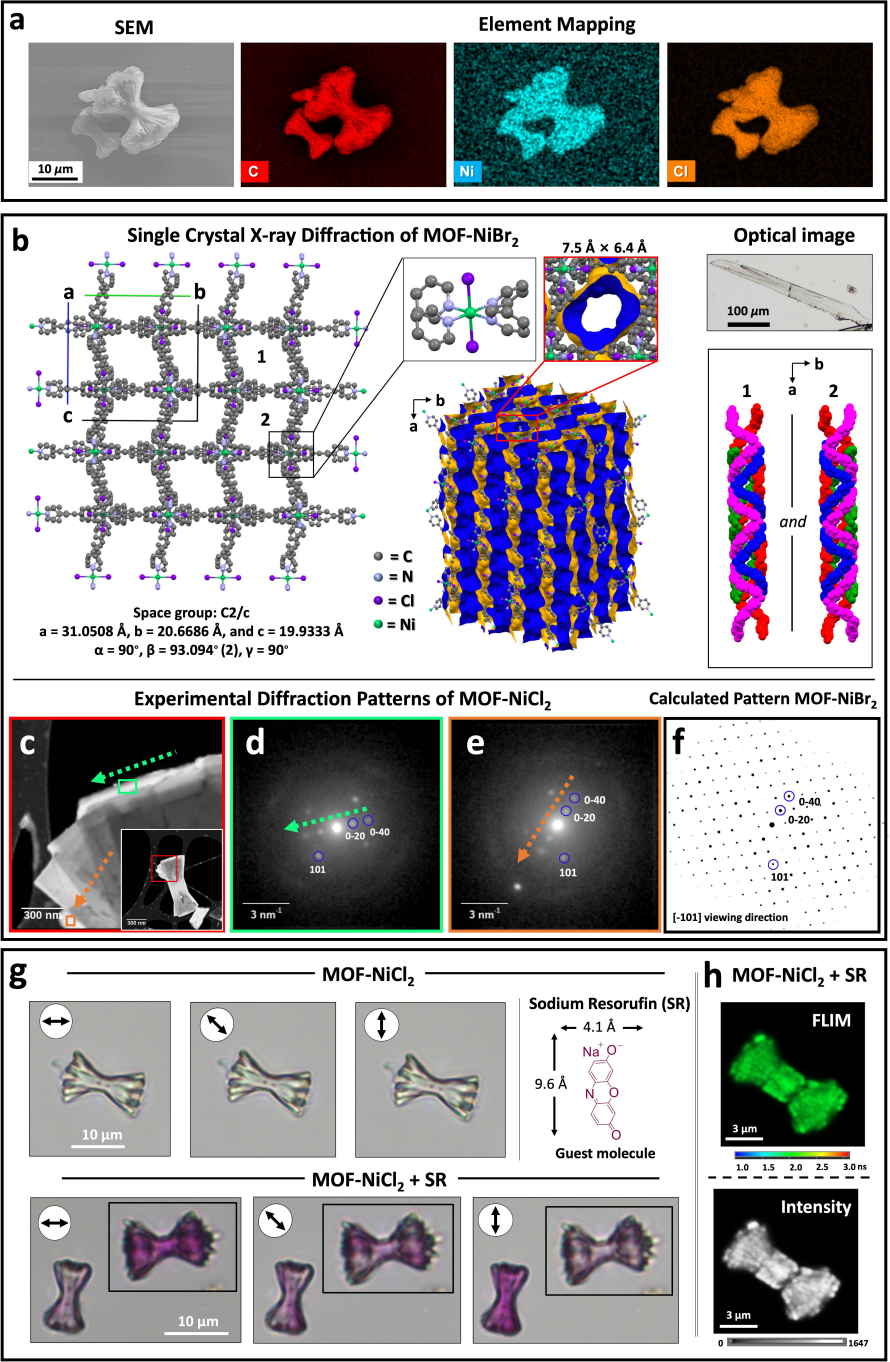
(a) Scanning electron microscopy (SEM) image and corresponding energy‐dispersive X‐ray spectroscopy (EDS) elemental maps of **MOF‐NiCl_2_**. (b) Single‐crystal X‐ray structure of **MOF‐NiBr_2_** (CCDC 2255298).^[44]^ The optical microscopy image shows the crystal of **MOF‐NiBr_2_** used for single‐crystal X‐ray diffraction (SCXRD). (c–e) Scanning nanobeam electron diffraction of sheaf‐like **MOF‐NiCl_2_**. (c) Virtual dark‐field image of a fan‐like tip. The inset shows a lower magnification image of the whole structure. (d, e) Region‐of‐interest diffraction patterns cumulated over the rectangular regions marked in (c). (f) Matching kinematical zone‐axis diffraction patterns calculated from the SCXRD data shown in (d) of **MOF‐NiBr_2_**_,_ with the diffraction patterns (d) and (e) of **MOF‐NiCl_2_**. (g) Optical microscopy images of **MOF‐NiCl_2_** (top) and **MOF‐NiCl_2_** functionalized with sodium resorufin (**SR**) (bottom). (h) Confocal fluorescence lifetime imaging microscopy (FLIM) (top) and intensity images (bottom) of **MOF‐NiCl_2_** functionalized with **SR**.

Energy‐dispersive X‐ray spectroscopy (EDS) combined with SEM showed that the elemental composition of the sheaf superstructures (major product) and the rods (minor product) are similar (Figures 2a, S3). The different components: carbon (from the ligand), nickel cations and chloride anions are homogeneously distributed. Micro‐Raman spectroscopy revealed that the vinyl‐pyridine moieties in both types of morphologies coordinate to the metal centers (Figures S4a–b). The intensities of the pyridine ring bands of the ligand at *ν*=992 cm^−1^ and 1001 cm^−1^ are blue shifted to *ν*=1008 cm^−1^ and 1025 cm^−1^, respectively, as expected for the formation of pyridine‐Ni^2+^ complexes.^[30]^ These observations imply that the sheaf superstructures (major product) and the rods (minor product) are structurally equivalent. The composition and the coordination chemistry were confirmed by X‐ray photoelectron spectroscopy (XPS) of the bulk (Figure S4c). The nickel cations retained their oxidation state (+2). Two distinctive peaks at 855 eV (2p_3/2_) and 872.5 eV (2p_1/2_), and the satellite bands (for 2p_3/2_=859–869 eV and 2p_1/2_=876–886 eV) can be attributed to the 2p orbitals of Ni. The signal for N is present at 400 eV (1 s), and the bands for the counterions Cl (−1) were observed at ~199 eV (2p_3/2_). For comparison, the signal of the nitrogen atoms of the free ligand appears at 399 eV (1 s), and for Ni of NiCl_2_ at 856 eV (2p_3/2_) and 873.5 eV (2p_1/2_). These shifts are indicative of the coordination of the vinyl‐pyridine moieties to the nickel cations.^[31]^

The crystals of **MOF‐NiCl_2_** were too small to analyze by single‐crystal X‐ray diffraction (SCXRD). Therefore, other nickel salts were used to grow larger crystals with an equivalent crystallographic structure. The ligand (**CSB**) was reacted with NiBr_2_, NiI_2_, Ni(NO_3_)_2_, and Ni(OAc)_2_ using identical reaction conditions as applied with NiCl_2_ (0.6 mM of **CSB** in CHCl_3_ and 1.2 mM of NiX_2_ in DMF). In line with our previous studies,[^27^, ^28^] we found that the morphology and size of the MOFs is highly dependent on the nickel salt used. Optical and SEM imaging show the formation of completely different morphologies (Figures S5, S6). Crystals obtained with NiBr_2_ were suitable for structural determination by SCXRD. The SCXRD analysis unambiguously showed the formation of a MOF (**MOF‐NiBr_2_**; Figure 2b). The simulated powder X‐ray diffraction (PXRD) of **MOF‐NiBr_2_** matches well with the experimental PXRD spectra of both **MOF‐NiBr_2_** and **MOF‐NiCl_2_** (Figure S7). Micro‐Raman spectroscopy further confirmed the structural similarity (Figure S4b) of these crystals that have strikingly different morphologies. The crystal structure of **MOF‐NiBr_2_** is centrosymmetric monoclinic *C*2/c space group (a=31.0508 Å, b=20.6686 Å, c=19.9333 Å, *β*=93.094°, CCDC 2255298, Table S1).^[44]^ The 3D framework is formed by coordination of four pyridine moieties from four different **CSB** ligands and two Cl anions with the bivalent metal center. The six ligands and the metal cation form octahedral geometry. The pyridine moieties of the achiral ligand are arranged in a propeller‐type, chiral arrangement, possibly to minimize the steric hindrance between adjacent ligands.^[32]^ The four Ni−N distances of 2.090 Å, 2.094 Å, 2.096 Å, 2.108 Å, and those of Ni–Cl (2.493 Å) are well within the ranges normally found for NiX_2_(pyr)_4_ (X=halide). The structure has continuous nanochannels that run in parallel to the *a*‐axis. Thepore cross section of the nanochannels is 7.5 Å×6.4 Å and the framework contains 41 % solvent accessible voids of the total volume (calculated by Mercury CSD 2020.1.1 using a spherical probe with a radius of 1.2 Å).

The nanobeam electron diffraction (NBED) patterns of selected areas of sheaf‐type **MOF‐NiCl_2_** consist of well‐defined sharp spots indicating single‐crystallinity (Figures 2c–e, S8a–d). The analyzed fantail nanorods are isomorphous crystals. However, the spatial orientation (based on the electron diffraction data) of individual nanorods deviates slightly (~3°) from each other. Such a minor deviation in the spatial orientation between neighboring nanorods is typical for superstructures formed by a non‐crystallographic branching mechanism.[^1^, ^33^] This misorientation results in the overall polycrystalline character of the sheaf‐like superstructures. The electron diffraction from the free bridge was opaque for electrons, and no information could be obtained about the crystallographic properties. Therefore, diffraction data was collected at the thinner edges. **MOF‐NiCl_2_** and **MOF‐NiBr_2_** are isomorphous as indicated by the symmetry match of the NBED for **MOF‐NiCl_2_** with zone‐axis patterns calculated from the refined structure of **MOF‐NiBr_2_** (Figure 2f).

This observation is in good agreement with the PXRD data. By indexing hkl reflections we can infer that the orientation of the nanorods is correlated with their shape. The long direction of the rods runs along the *a*‐axis and the cross‐section of the rods is the *bc*‐plane. The plane of the metal atoms on the *b*‐axis is parallel to one of the side faces and the radial fan direction is coincident with the <101> direction of the nanorods. The channels along the *a*‐axis are inclined with respect to radial fan direction. The diffraction patterns of the minor rod‐like product of **MOF‐NiCl_2_** (Figures S8e–h) show that these structures are isostructural with both the sheaf‐like crystals of **MOF‐NiCl_2_** and with **MOF‐NiBr_2_**.

To demonstrate the sieving properties and molecular functionalization of the crystals, we suspended a batch of **MOF‐NiCl_2_** for one hour in an ethanol solution of sodium resorufin (**SR**, 8.9×10^−5^ M). This chromophore has excellent fluorescent properties and can easily enter the channels of the structures (dimensions of **SR**: 4.1 Å×9.6 Å; cross‐section of the channel is 7.5 Å×6.4 Å). The negative charge of the **SR** dye facilitates its uptake by the cationic framework. The initial **MOF‐NiCl_2_** is colorless, also under polarized light (Figure 2g, top). The optical images of isolated **SR**‐functionalized crystals clearly show the homogeneous distribution of the dye, in the free bridge as well as in the fantails nanorods (Figure 2g, bottom). This observation demonstrates that the channels are chemically accessible regardless of their different spatial orientation. The intensity of the purple color shows pronounced changes at different angular directions, under polarized light which strongly supports the alignment of dye molecules inside the channels. The polycrystalline nature of the MOFs results in interesting optical properties. The individual sheaf‐like crystals have areas that color differently under polarized light. When the polarized light is orientated parallel to the central rod, the crystals are purple. The crystals are slightly colored when the light source is positioned perpendicularly. Other incident angles result in different coloration intensities of the different areas of the sheafs. Confocal fluorescence lifetime imaging microscopy (FLIM) of crystals loaded with **SR** were obtained by exciting at *λ*=570 nm and detecting the emission at *λ*=650–700 nm (Figure 2h). The fluorescence lifetime of the functionalized sheafs and nanorods (=minor product) are similar (≈1.8 ns), indicating the same arrangement of the dyes inside the channels (Figure S9). These nanorods also showed a homogeneous distribution of the dye and polarized light‐dependent optical properties.

**Figure 3:**
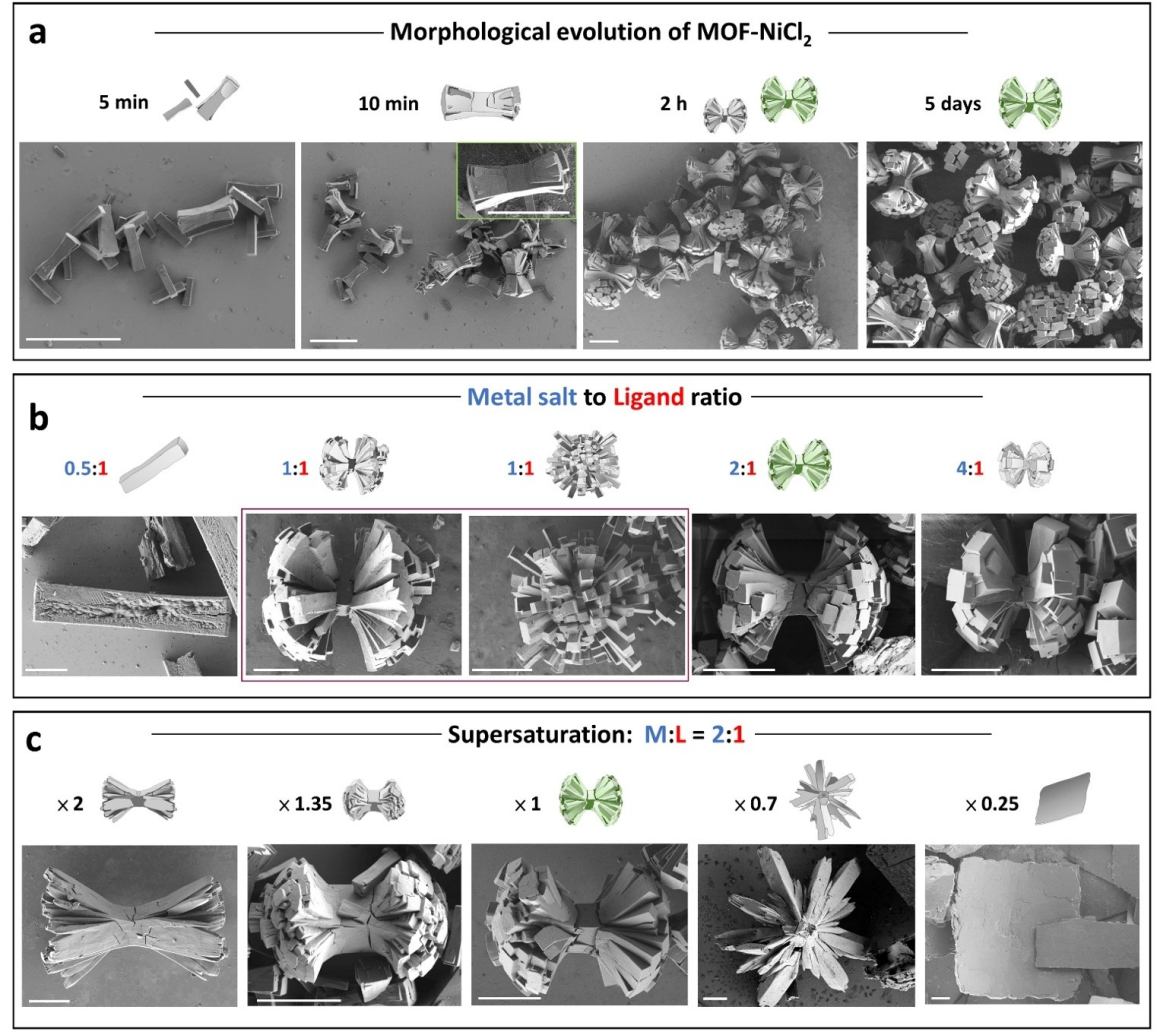
(a) Time‐dependent scanning electron microscopy (SEM) images showing the formation of the sheaf‐like **MOF‐NiCl_2_**. Conditions: 105 °C, NiCl_2_:**CSB**=2 : 1, **CSB**=0.6 mM. Scale bar=10 *μ*m. (b, c) SEM images showing the structures formed by varying the (b) metal salt‐to‐ligand molecular ratios and (c) using different concentrations with the same metal salt‐to‐ligand molecular ratio of 2 : 1.

The formation of the sheaf‐like superstructures was followed by *ex‐situ* SEM measurements. The solvothermal reaction was stopped at different reaction times (*t*=5 min to *t*=5 days; Figure 3a). No precipitation was observed for *t*<5 min. Structures having a rod‐like morphology (length=4.0±0.7 *μ*m) were observed at *t*=5 min. Branches are present on some of the structures and have a smaller width than the rods. The branching is evident from the visible lines where the new structures grew. Electron diffraction showed that these rods already have crystalline packing similar to the sheafs that are formed after 5 days (Figure S10). After *t*=10 min, we observed the formation of hedrites; hyperbranching is clearly seen on both ends giving an appearance of fantail structures. The size of the fantails increases with time and well‐defined sheaf‐like superstructures develop from the hedrites at *t=*2 h. As the reaction time increases (*t*=5 days) no significant changes in the morphology of the crystals were observed, and no further branching occurred to form spherulites; under these conditions the sheafs are the thermodynamic product.

To understand the formation of our MOFs in more detail, the stoichiometry and the supersaturation of the building blocks were varied to control the growth kinetics and concentration of nuclei formation during crystallization (Figures 3b, c). The morphology and size of the crystals changed with the stoichiometry of the building blocks. The ratios of the two building blocks, NiCl_2_:**CSB**, were varied within the range 0.5 : 1 to 4 : 1. The starting concentration of **CSB** was 0.6 mM for these experiments. We observed large rod‐like crystals (*l*=40.4±10.2 *μ*m) when using a metal salt‐to‐ligand ratio of 0.5 : 1 (Figures 3b, S11). When the metal salt‐to‐ligand ratio is 1 : 1 both sheafs and spherulites are formed. These sheafs (36.2±6.1 *μ*m) are larger compared to the sheafs obtained when using a metal‐to‐ligand molar ratio 2 : 1 (12.0±2.5 *μ*m). Under this condition, the sheafs are metastable and continue to develop into the thermodynamically favorable spherulites (50.4±8.6 *μ*m). Almost all the branches of the fantails are directly attached to the nucleating central rod. It indicates that the surfaces of the central rods become unstable and non‐crystallographic branching occurs. The surfaces of the resulting square‐branches are more stable as is evident from the single generation of branching (Figures 3b, S12). These structures bear some similarity to fluorapatite‐gelatine reported by Kniep,^[3]^ and have only a first‐generation branching. Increasing the metal‐to‐ligand ratio to 4 : 1 produced mostly plates (18.6±3.5 *μ*m), half sheafs (12.3±1.1 *μ*m) and sheafs as a minor product (11.6±3.4 *μ*m) (Figures 3b, S13).

The relationship between the morphology and the concentration of the two building blocks was also evaluated (Figure 3c). In these experiments, the metal salt‐to‐ligand molar ratio of 2 : 1 was kept constant. However, the concentrations of both NiCl_2_ (1.2 mM) and **CSB** (0.6 mM) were varied by a factor of 2, 1.35, 0.7, and 0.25. The highest concentration (×2) resulted in large plates as the major product and hedrites (16.8±2.5 *μ*m) as a minor product (Figures 3c, S14). The branches are relatively thin and less prominent. Such structures are known to be precursors for the formation of sheafs. The concentration of ×1.35 resulted in the formation of well‐defined sheaf‐like crystals (9.5±1.6 *μ*m) with the free bridge having a length of 3.4±0.5 *μ*m and a width of 2.6±0.4 *μ*m. Along with sheaf‐like structures, nanorods were observed as a minor product (Figures 3c, S15). Decreasing the concentrations to ×0.7 generated plates (37.6±5.8 *μ*m) having a layered surface texture and larger structures (51.4±6.2 *μ*m) that resemble half of the sheafs (Figures 3c, S16). Some of these latter structures have hollow tubular sub‐units. Further decreasing the concentrations to ×0.25 resulted in smooth plates (55.8±7.2 *μ*m) (Figures 3c, S17). The structures formed under the abovementioned different reaction conditions (concentration and metal salt‐to‐ligand ratio) have similar homogeneous elemental compositions (EDS‐SEM) and Ni‐pyridine coordination chemistry (XPS) (Figures S18–S21). All structures have a metal cation‐to‐ligand ratio of 2 : 1, therefore all the reaction solutions contained excess of the metal salt but in different concentrations. The metal cations modulate the crystal morphology, dimensions and their uniformity.

## Conclusions

In conclusion, the complex morphology changes result in superstructures with retention of their initial crystallinity without the need for external modulators or additives. An excess of one of the molecular building blocks results in the saturation of the crystal surface and trapping of the other compound in solution. Both processes are expected to decrease the rate of the crystal growth. In an anisotropic structure like ours, this can lead to preferable binding of the excess molecular component to the growing facets, thereby altering the crystal habit. Therefore, instead of using an “external” modulator with a chemical structure different than that of the molecular building blocks, the building blocks can be used as an “internal” modulator simply by changing their molecular ratios. Tailor‐made additives are well known for shaping crystals.^[34]^ Alivisatos, Kniep, Yang, and others reported the use of splitting agents.[^3^, ^4^, ^35^, ^36^, ^37^, ^38^] Sheaf‐like antimony sulfide crystals were obtained by using different quantities of a copolymer to control the level of splitting.^[37]^ In contrast to the splitting mechanism often observed for other materials, this study shows that hyperbranched MOFs can form by non‐crystallographic branching.

We found that synthetic parameters such as concentration and metal salt‐to‐ligand ratio and different coordinative counter anions (Br^−^, I^−^, NO_3_^−^, and OAc^−^), play an important role in shaping the size and morphology of our materials. As a result, a series of different structures resembling rods, sheafs, spherulites, and plates were obtained. We hypothesize that the hyperbranching is caused by an increasing number of defects on the initially fast grown rods. After a certain time, the basal facets become less reactive and instead material is being added to the prism facets. The rate of the secondary nucleation and subsequent crystal growth (=branching) is reflected in the formation of the triangular “branches”. These initial branches are fully connected to the surface of the rod. Continuous growth in the lateral direction results in square branches longer than the central slower growing rod. The here reported crystals are the first of such MOF superstructures and the apparent branching mechanism can explain some of the crystal shapes formed with other materials. They are certainly unusual within this class.[^39^, ^40^, ^41^] In spherulites, narrow fibrils are often formed because of competition and geometric constraints.^[1]^ Here, each crystal competes by growing larger faces, not fading into a collective behavior. Our finding could be extended to other coordination materials so that similar and new branched morphologies can be obtained with new optical properties.[^42^, ^43^]

## Supporting Information Summary

Experimental details, materials and methods, additional characterization data. Deposition Number CCDC 2255298 contain the supplementary crystallographic data for this paper. These data are provided free of charge by the joint Cambridge Crystallographic Data Centre and Fachinformationszentrum Karlsruhe.

## Conflict of Interests

The authors declare no conflict of interest.

1

## Supporting information

As a service to our authors and readers, this journal provides supporting information supplied by the authors. Such materials are peer reviewed and may be re‐organized for online delivery, but are not copy‐edited or typeset. Technical support issues arising from supporting information (other than missing files) should be addressed to the authors.

Supporting Information

Supporting Information

## Data Availability

The data that support the findings of this study are available in the supplementary material of this article.
