# Supplementary material for: Morphological Evolution of Metal‐Organic Frameworks into Hedrite, Sheaf and Spherulite Superstructures with Localized Different Coloration
Source: Chemistry. 2024 Dec 13;31(7):e202403577. doi: 10.1002/chem.202403577 (PMC11789549; doi:10.1002/chem.202403577)
Supplement: Supplementary file 1 — Supporting Information [file CHEM-31-e202403577-s001.pdf]

Chemistry–A European Journal

Supporting Information

Morphological Evolution of Metal-Organic Frameworks into Hedrite, Sheaf and Spherulite Superstructures with Localized Different Coloration

Naveen Malik, Linda J. W. Shimon, Lothar Houben, Anna Kossoy, Iddo Pinkas, Ifat Kaplan-Ashiri, Tatyana Bendikov, Michal Lahav,* and Milko E. van der Boom*

Supporting Information

Morphological Evolution of Metal-Organic Frameworks into Hedrite, Sheaf and Spherulite Superstructures with Localized Different Coloration

Naveen Malik,[a],[b] Linda J. W. Shimon,[c] Lothar Houben,[c] Anna Kossoy,[c] Iddo Pinkas,[c] Ifat Kaplan Ashiri,[c] Tatyana Bendikov,[c] Michal Lahav,*,[a] and Milko E. van der Boom*,[a]

[a] Department of Molecular Chemistry and Materials Science, Weizmann Institute of Science, 7610001 Rehovot, Israel. Email: michal.lahav@weizmann.ac.il; milko.vanderboom@weizmann.ac.il

[b] Department of Chemistry, College of Engineering and Technology, SRM Institute of Science and Technology, Kattankulathur 603203, India.

[c] Department of Chemical Research Support, Weizmann Institute of Science, 7610001 Rehovot, Israel.

EXPERIMENTAL SECTION

Materials and Methods

Glass pressure tubes (Ace Glass, Inc., pressure tubes #15 with a plunger valve, PTFE Bushing and FETFE® O-Ring, volume 50 mL) were cleaned by immersion in concentrated H_2SO_4 for 24 h and washed with deionized water and then dried for 24 h at 130°C. Chloroform ($CHCl_3$ ≥ 99.8%) and dimethylformamide (DMF ≥ 99.8%) were purchased from Bio-Lab and Sigma Aldrich, respectively. Reagents, metal salts ($NiCl_2·6H_2O$, $NiBr_2·\times H_2O$, NiI_2, $Ni(NO_3)_2·6H_2O$, $Ni(OAc)_2·4H_2O$) and sodium resorufin (**SR**) were purchased from Merck and Sigma-Aldrich. Reagents were used without further purification. Tetrakis(4-(2-(pyridin-4-yl)ethyl)phenyl) methane (**CSB**) was prepared according to a literature procedure.[S1]

Preparation of the metal-organic frameworks (MOFs)

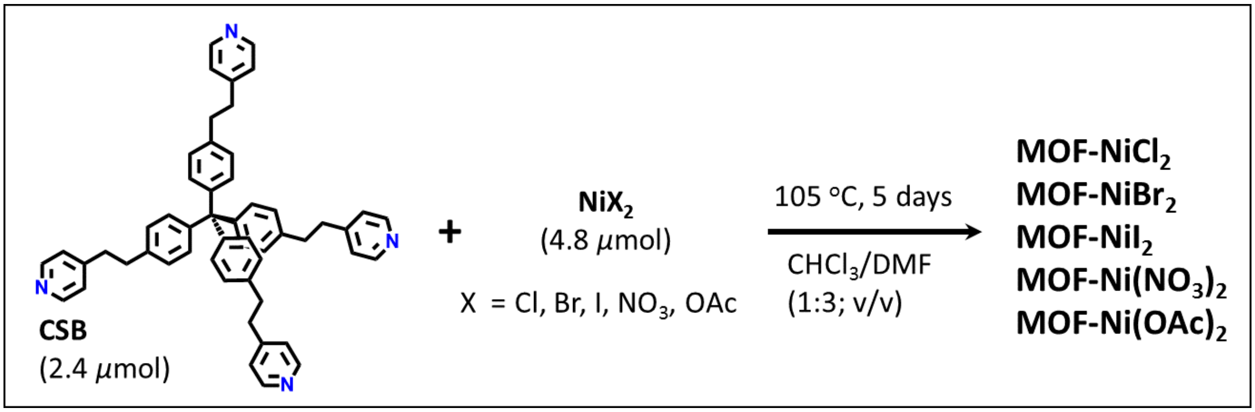

A $CHCl_3$ solution (1.0 mL) of tetrakis(4-(2-(pyridin-4-yl)ethyl)phenyl) methane (**CSB**, 1.8 mg, 2.4 μmol)[S1] was added to a DMF solution (3.0 mL) containing $NiCl_2·6H_2O$, (1.1 mg, 4.8 μmol, 2 eq.), in a glass pressure tube. The concentrations of **CSB** and $NiCl_2·6H_2O$ are 0.6 mM and 1.2 mM, respectively. The tube was sealed and heated at 105 °C in an oven for 5 days. The thermostat of the oven was lowered with 10 °C every hour, until the temperature reached 25 °C. The MOFs were isolated by centrifugation (3500 rpm, 4 min). The solvent was discarded, and the MOFs were washed with DMF, $CHCl_3$, and EtOH. Drying the MOFs under high vacuum (≈ 10^{-2} mbar) for 12 h afforded a white powder (95% yield based on **CSB**). Identical procedures for the formation of MOFs with $NiBr_2$, NiI_2, $Ni(NO_3)_2$ and $Ni(OAc)_2$ were used. The yields based on **CSB** are > 80%.

Metal salt-to-ligand molar ratio – morphology relationship for MOF-$NiCl_2$

MOF-$NiCl_2$ were prepared under similar reaction condition as described above. The amount of ligand (**CSB**) was kept constant (2.4 μmol). The metal salt-to-ligand molar ratio is 0.5:1, 1:1, 2:1 and 4:1 (**Figure S11-S13**).

Concentration – morphology relationship for MOF-$NiCl_2$

The procedure is similar to that described above. The molar ratio of the metal salt-to-ligand was kept constant at 2:1. The amounts of ligand (**CSB**) used: 4.8 μmol, 3.2 μmol, 2.4 μmol, 1.7 μmol and 0.6 μmol (**Figure S14-S17**).

Scanning electron microscopy (SEM)

SEM measurements were performed using HRSEM ULTRA-55 ZEISS and HRSEM SUPRA-55 VP ZEISS instruments at landing voltage of 1.5 kV. Images of secondary and backscattered electron modes were collected using the Everhart-Thornley and energy selective backscattered detectors, respectively. The SEM-energy-dispersive X-ray spectroscopy (EDS) mapping was performed by using a four-quadrant retractable EDS detector (Bruker FlatQUAD) at accelerating voltages of 12 kV. The aperture size was set to 30 microns. Samples were prepared by placing a drop of the reaction mixture on a silicon substrate and the solvent was allowed to evaporate. The surface of the sample was coated with a 5 nm-thick iridium layer.

Functionalization of MOF-$NiCl_2$ with sodium resorufin (SR)

MOF-$NiCl_2$ (~2 mg) was washed with ethanol (2×) to remove residual DMF. Subsequently, the crystals were transferred to an Eppendorf tip, and 200 μL of an ethanol solution containing sodium resorufin (8.9×10^{-5} M) was added. After 1 h, the solution was discarded, and the crystals were washed with ethanol (2×). Optical images of the functionalized crystals were captured on a glass slide.

Optical Microscopy

Light microscopy images were obtained with Nikon's Eclipse E600 and Nikon Eclipse LV100ND microscopes. The latter microscope is equipped with DeltaPix 2001-2022 v6.5.3 software that allows for extended focus imaging.

Fluorescence lifetime imaging microscopy (FLIM)

Fluorescence lifetime and intensity images of crystals loaded with sodium resorufin were obtained using a Leica TCS SP8 STED equipped with a tunable (470−670 nm) pulsed white light laser (WLL; repetition rate = 78 MHz) (Leica Microsystems). A sample of crystals loaded with sodium resorufin was deposited on a glass slide and covered with a cover slide. The magnetization of the objective is HC PL APO CS2 20×/0.75 DRY. The functionalized crystals were excited at λ = 570 nm and the fluorescence signals were collected λ = 650-700 nm. The pixel size was set to 0.191 μm. The final image size was 97.72 μm × 97.72 μm. The laser power was adjusted (0.02% - 0.07%) to avoid signal saturation. All analyses were done with Leica Microsystem software.

Transmission Electron Microscopy (TEM)

A suspension (10 μL) of as-synthesized crystals was dispersed on lacey carbon support on molybdenum grids for TEM analysis. Scanning nanobeam electron diffraction (NBED) data were obtained in a double-aberration corrected TFS Themis-Z microscope, equipped with a high-brightness FEG source at an acceleration voltage of 200 kV. For the 4D-STEM scanning nanobeam recording, the sample was kept at liquid N_2 temperature in a Gatan 914 cryo-holder (Gatan Inc., Pleasanton/CA, USA) to avoid radiation damage to the crystals. The hybrid pixel electron microscope pixel array detector (EMPAD) allowed rapid data collection of entire unsaturated diffraction patterns with a pixel dwell time of 1 ms for each pattern. An electron probe with a convergence angle of 0.2 mrad was adjusted in the STEM microprobe mode, further defocused to a probe size of a few 10 nm in diameter to reduce electron flux. A primary beam current of less than 4 pA was used. Typically, scanning diffraction data sets were acquired over a spatial raster of 128 × 128 pixels. The total exposure in the 4D-STEM experiments was approximately 1 e/Å^2.

X-ray Photoelectron Spectroscopy (XPS)

XPS measurements were carried out by Kratos AXIS ULTRA system using a monochromatic Al Kα X-ray source (hν = 1486.6 eV) at 75W and detection pass energies ranging between 20 and 80 eV. A low-energy electron flood gun (eFG) was applied for charge neutralization. To define the binding energies (BE) of different elements the C1s line at 284.8 eV was taken as a reference.[S2, S3] Curve fitting analysis was based on linear or Shirley background subtraction and application of Gaussian-Lorentzian line shapes.

Raman spectroscopy

Raman measurements were conducted on a LabRAM HR Evolution instrument (Horiba, France) configured with four laser lines (325 nm, 532 nm, 632 nm, and 785 nm), allowing for Raman spectra from 50 cm^{-1} and onward. The instrument is equipped with an 800 mm spectrograph allowing for high spectral resolution and low stray light. The system's pixel spacing is 1.3 cm^{-1} when working with a 600 grooves/mm grating at 632.8 nm excitation; it is also equipped with other gratings, allowing for lower and higher spectral resolution. The sample is exposed to light by various objectives (MPlanFL N NA-0.9, 100X, LUMPLFLN NA-1.0 60XW, and LMPlanFL N NA-0.5 50X LWD, Olympus, Japan). The LabRAM instrument is equipped with a CCD detector: a 1024 × 256 pixel open electrode front illuminated CCD camera cooled to −60 °C. The system is set around an open confocal microscope (BX-FM Olympus, Japan) with a spatial resolution better than 1 μm using the 100X objective. The sample is placed upon a motorized stage that can be software-controlled to measure the Raman spectral maps of the samples. The measurements were performed using a 632.8 nm HeNe laser, with 600 grooves/mm grating and a 100X objective.

X-ray crystallography

A single crystal of **MOF-$NiBr_2$** suitable for X-ray diffraction was coated with Paratone® oil (Hampton Research, CA, USA), mounted on a MiTeGen loop and flash frozen in liquid nitrogen. Diffraction data were recorded on Rigaku Synergy R system equipped with a HyPix-Arc 150° detector. Data were measured with CuKα radiation at 100(2) K. The data were collected and processed with Rigaku OD 'CrysAlisPro 1.171.42.74a (Rigaku OD, 2022)'. The structure was determined by direct methods using SHELXT-2018 with SHELXL-2019/2 and SHELXL-2019/2.[S4-S6] The crystal data and the structural refinement are summarized in **Table S1**.

Table S1. Crystal data and structure refinement of MOF-$NiBr_2$.

Formula	$C_{53}H_{48}Cl_2N_4Ni$	
Crystal Description	Needle	
CCDC	2255298	
Formula weight	997.47 (g/mol)	
Temperature	100 K	
Wavelength	1.54184Å	
Crystal system	Monoclinic	
Space group	*C*2/*c*	
Unit cell dimensions	a = 31.0508(9) Å	α = 90°
	b = 20.6686(10) Å	β = 93.094°
	c = 19.9333(5) Å	γ = 90°
Volume	12774.1(6) $Å^3$	
Z	8	
Density (calc)	0.905 g/cm^{-3}	
Absorption coefficient	1.423 mm^{-1}	
F (000)	3648	
Theta range for data collection (°)	3.346 to 66.595	
Reflections collected (Unique)	59749 (10625)	
R_{int}	0.0453	
Completeness (%)	94	
Data/restraints/parameter	10625/51/543	
Goodness-of-fit on F^2	1.294	
Final R [I>2σ(I)]	R_1 = 0.0915, wR_2 = 0. 2954	
R (all data)	R_1 = 0.1021, wR_2 = 0.3097	
Largest diff peak and hole	0.645 e·$Å^{-3}$ and -0.421 e·$Å^{-3}$	

Powder X-ray diffraction

The measurements were performed on a SmartLab (Rigaku, Japan) diffractometer equipped with a rotating Cu anode operating at 45 kV and 200 mA and with a HyPix-3000 two-dimensional detector. A quasi-parallel X-ray beam was formed by a multilayer mirror (CBO attachment, Rigaku), and shaped by a 2.5 degrees soler slit. Another 2.5 degrees soler slit was placed after the sample. Crystals with the mother solution were placed into a quartz capillary (⌀ = 0.5 mm, Capillary Tube Supplies LTS, UK) and centrifuged. Samples were scanned in the θ-2θ a mode with the detector in the 1D mode, the incident and receiving slits were 0.5 mm, the step was 0.01 degrees. Whole pattern simulations utilizing the Pawley method were made with MDIJade v8.8.

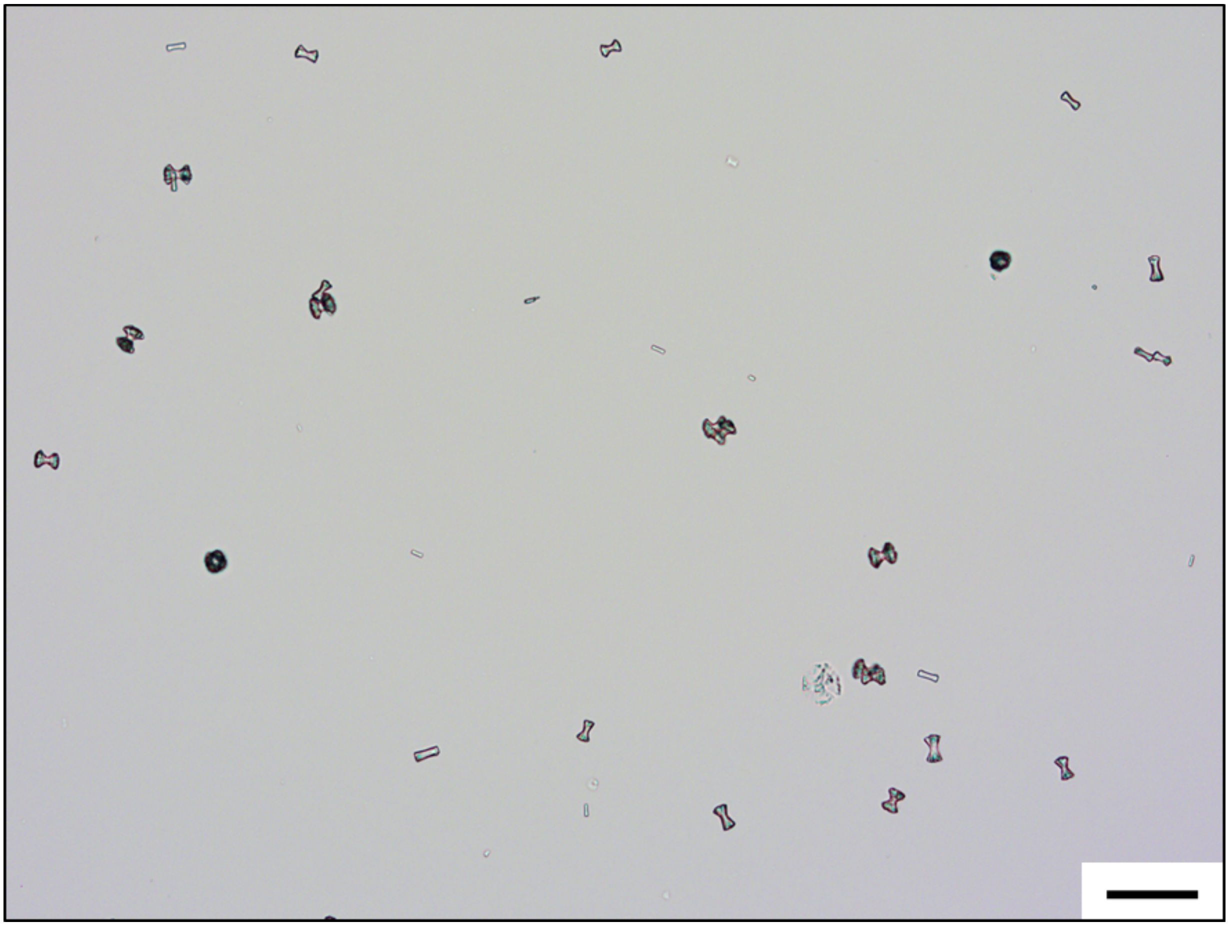

Figure S1. Representative optical microscopy image of sheaf-like **MOF-NiCl$_2$**. $NiCl_2 \cdot 6H_2O$ to **CSB** molar ratio = 2:1 with **CSB** = 0.6 mM. Scale bar = 50 μm.

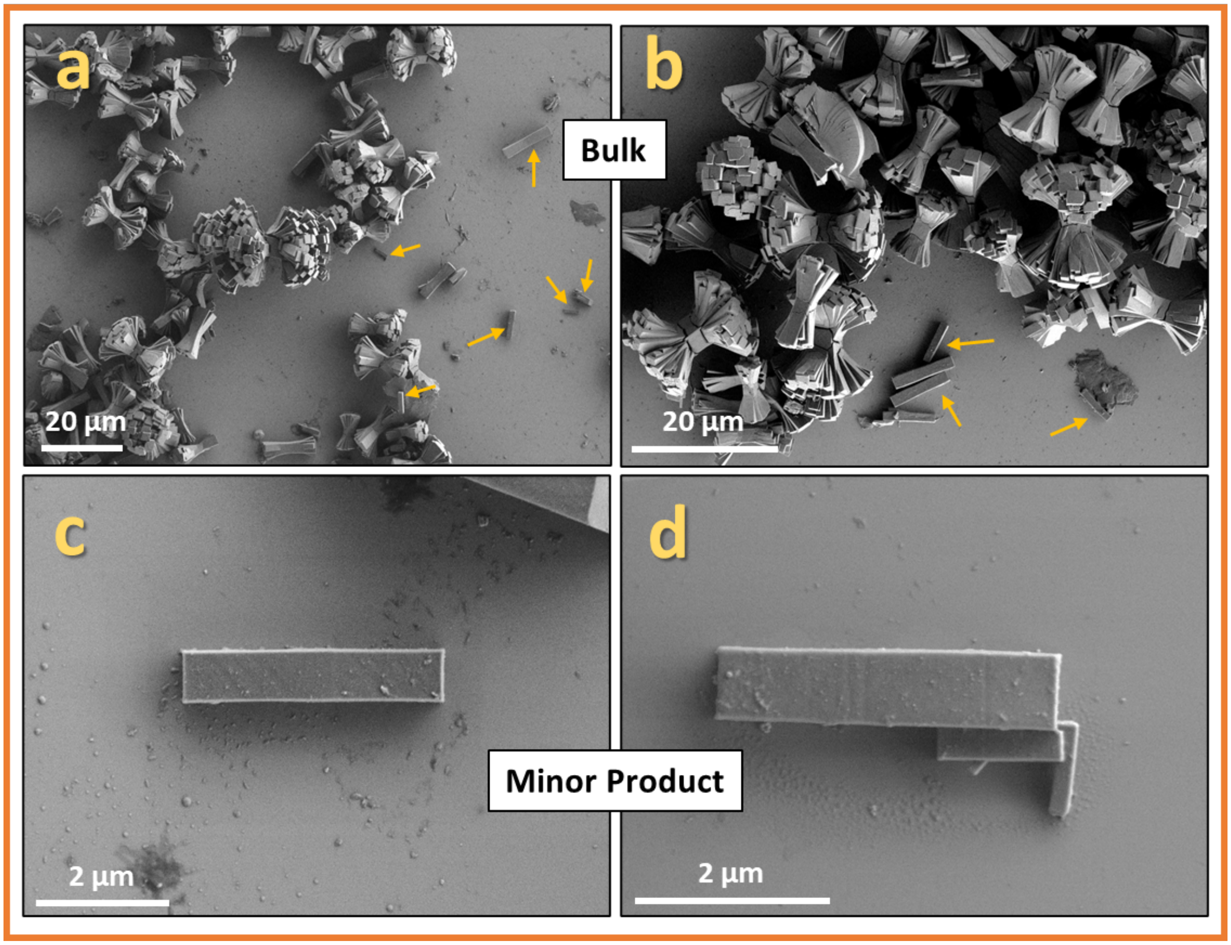

Figure S2. (a-d) Representative scanning electron microscopy (SEM) images of **MOF-NiCl₂** showing both sheaf- and rod-like morphologies. (c,d) The rod-like structures (also indicated by the yellow arrows in a and b) are observed as a minor product. The $NiCl_2 \cdot 6H_2O$ to **CSB** molar ratio = 2:1, with **CSB** = 0.6 mM.

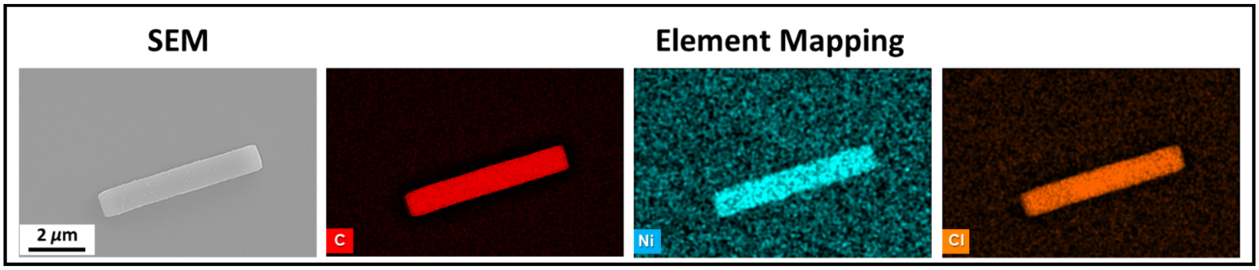

Figure S3. Scanning electron microscopy (SEM) and Energy-dispersive X-ray spectroscopy (EDS) intensity maps of rod-like **MOF-NiCl$_2$** (minor product; **Figure S2c,d**). The $NiCl_2 \cdot 6H_2O$ to **CSB** molar ratio = 2:1, with **CSB** = 0.6 mM.

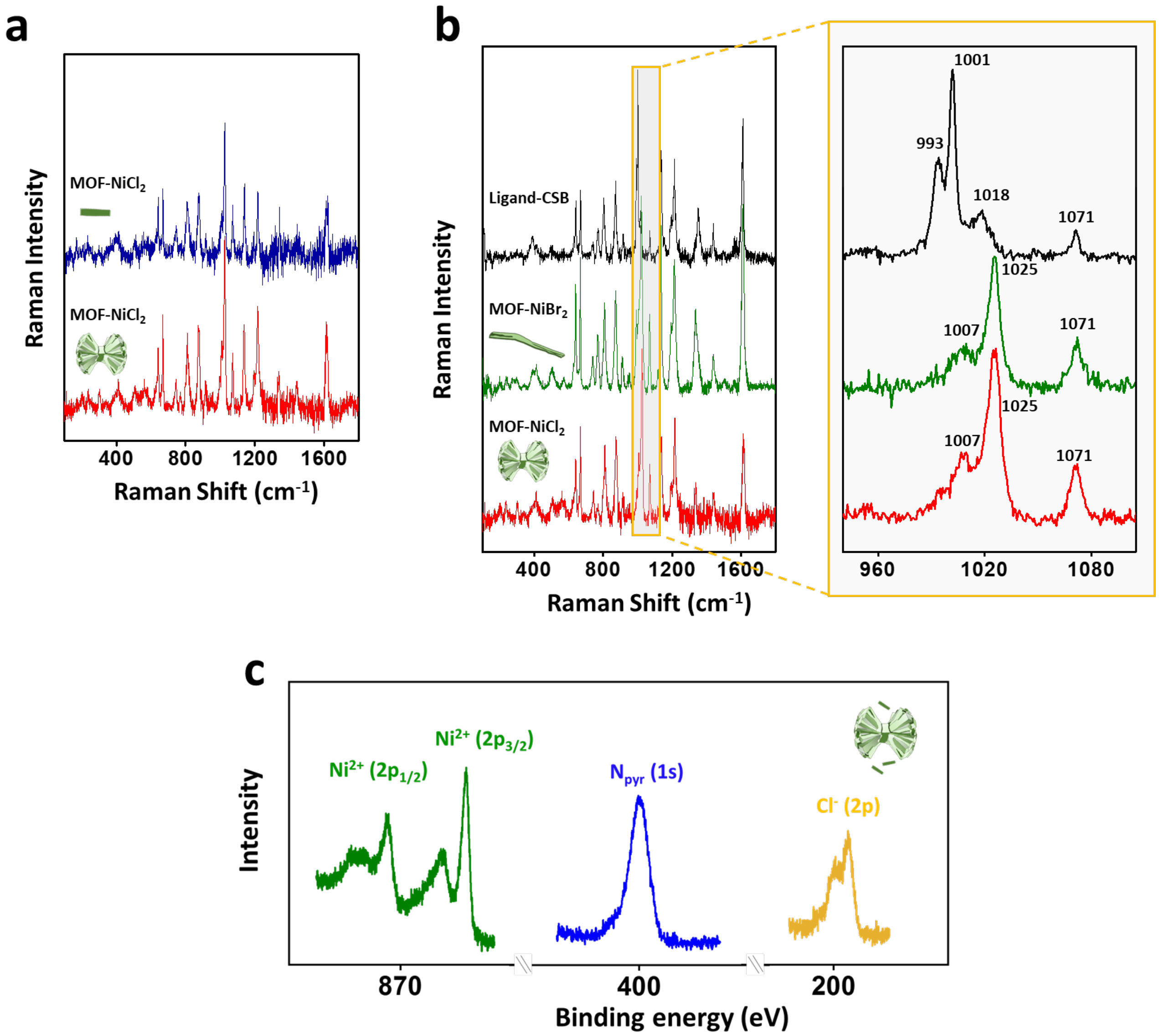

Figure S4. (a) Micro-Raman spectra of the rod- and sheaf-like morphologies of **MOF-NiCl₂**. (b) Micro-Raman spectra of the ligand (**CSB**), **MOF-NiBr₂**, and sheaf-like **MOF-NiCl₂**. The $NiBr_2 \cdot \times H_2O$ or $NiCl_2 \cdot 6H_2O$ to **CSB** molar ratio = 2:1 with **CSB** = 0.6 mM. (c) X-ray photoelectron spectroscopy (XPS) spectra of sheaf-like **MOF-NiCl₂**.

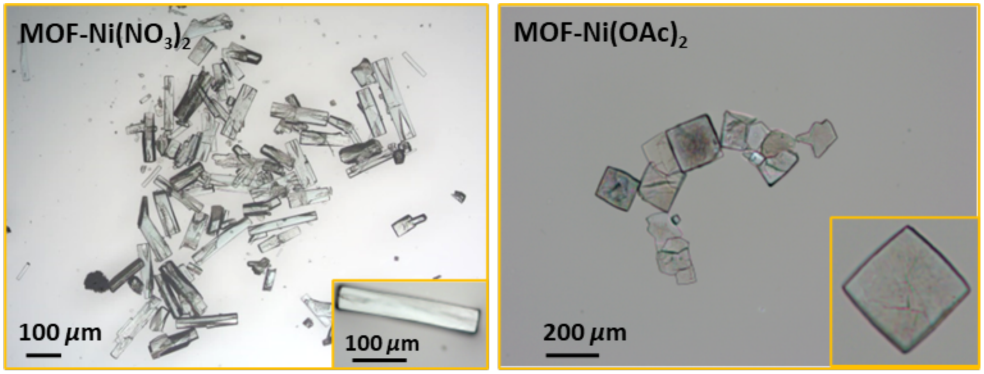

Figure S5. Representative optical microscopy images of **MOF-Ni(NO$_3$)$_2$** (left) and **MOF-Ni(OAc)$_2$** (right). The $Ni(NO_3)_2 \cdot 6H_2O$ or $Ni(OAc)_2 \cdot 4H_2O$ to **CSB** molar ratio = 2:1, with **CSB** = 0.6 mM.

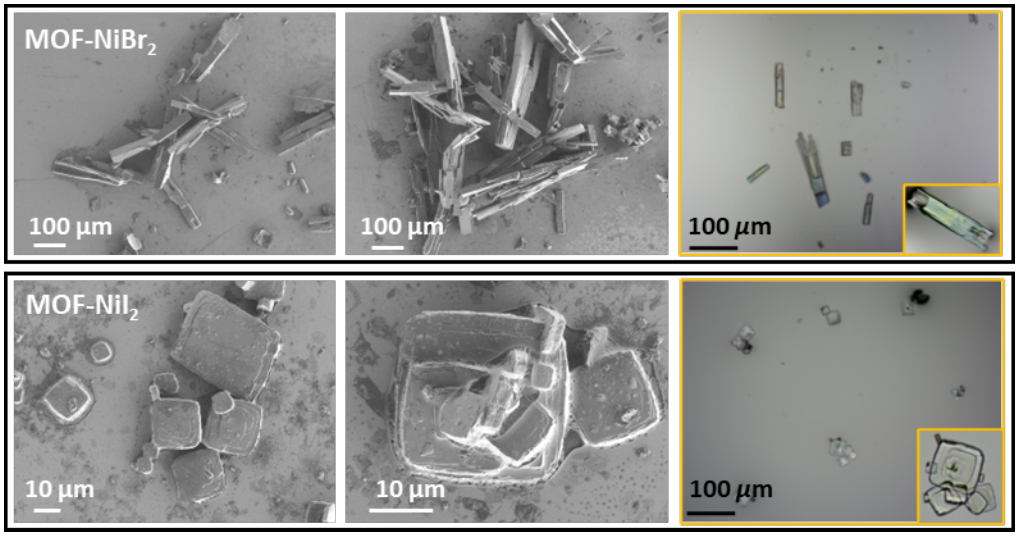

Figure S6. Representative scanning electron microscopy (SEM) and optical microscopy images of **MOF-NiBr$_2$** (top) and **MOF-NiI$_2$** (bottom). The $NiBr_2 \cdot \times H_2O$ or NiI_2 to **CSB** molar ratio = 2:1, with **CSB** = 0.6 mM.

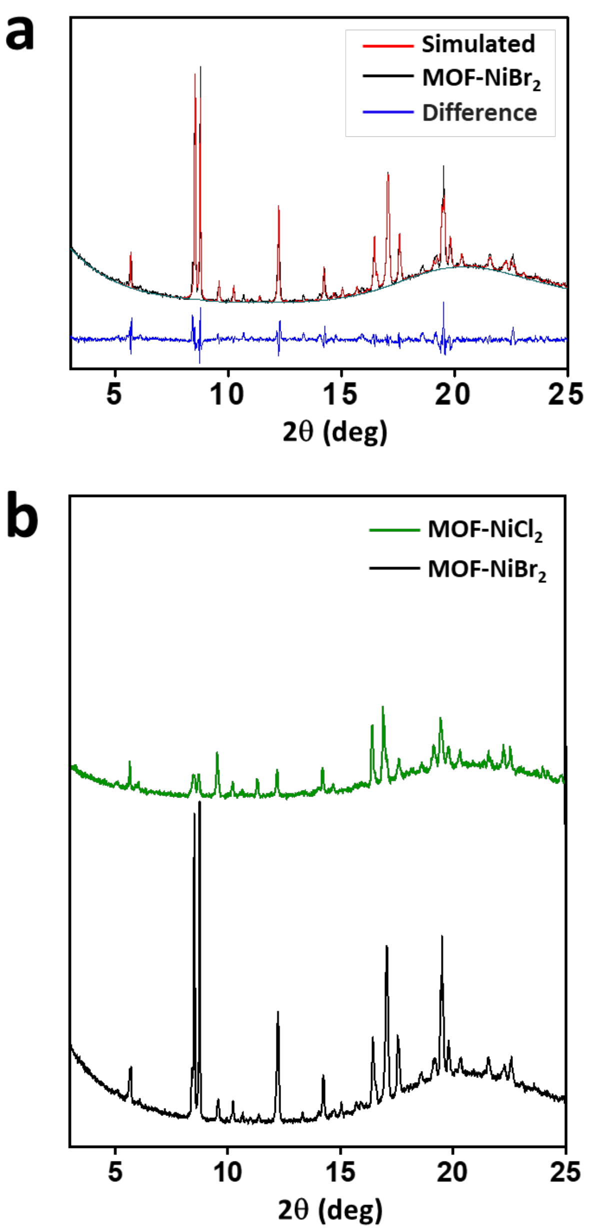

Figure S7. (a) Simulated (red) and experimental (black) powder X-ray diffraction (PXRD) pattern of **MOF-NiBr$_2$** and the difference (blue). The simulated pattern was generated by pattern indexing. The best fit was obtained for symmetry group P2/c(13) with lattice constants of 31.1144 Å, 20.24847 Å, 10.46887 Å and β = 91.419. This structure is closely related to the structure of CCDC 2255298 with the *c* parameter halved as a result of a slight distortion caused by the transition from 100K to RT. (b) Experimental PXRD patterns of sheaf-like **MOF-NiCl$_2$** (green) and **MOF-NiBr$_2$** (black). The $NiBr_2 \cdot \times H_2O$ or $NiCl_2 \cdot 6H_2O$ to **CSB** molar ratio = 2:1, with **CSB** = 0.6 mM.

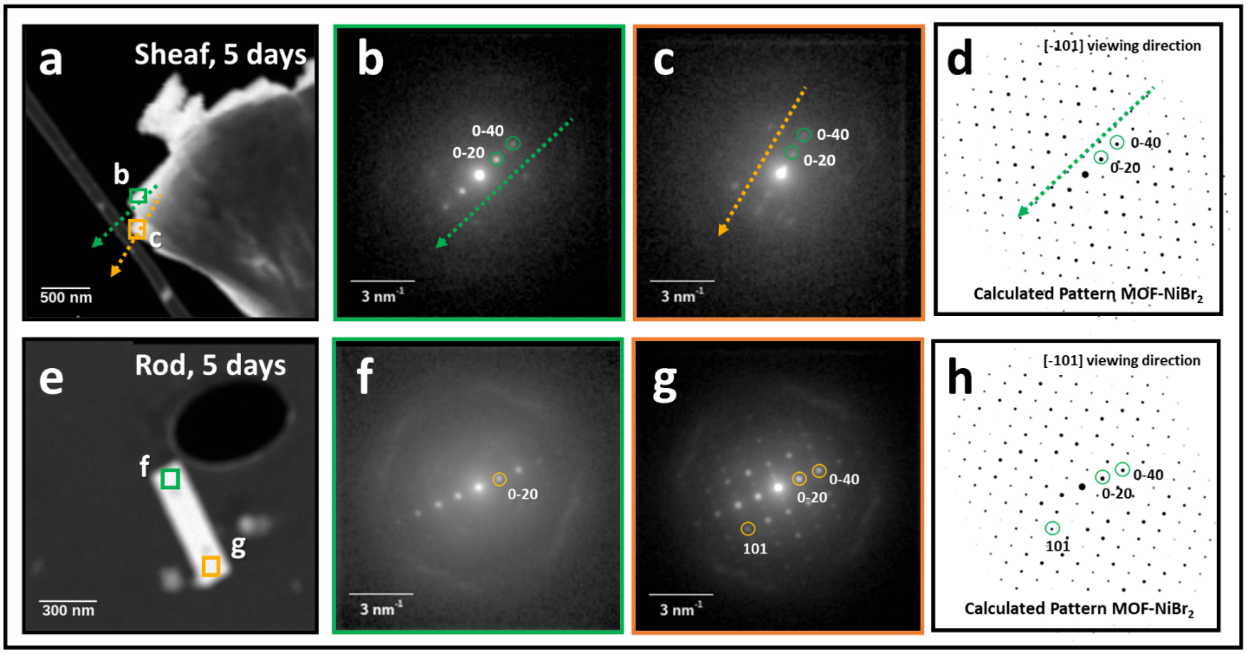

Figure S8. Scanning nanobeam electron diffraction (NBED). (a-c) Sheaf-like **MOF-NiCl₂** (major product). Corresponding diffraction data for two regions of interest marked are shown in (b) and (c). (d) Matching simulated electron diffraction (ED) obtained from the single-crystal data of **MOF-NiBr₂** (CCDC 2255298) with the data shown in (b). (e-g) Rod-like **MOF-NiCl₂** (minor product). (f) and (g) are sum diffraction patterns extracted for the two areas marked in (e). (h) Matching of the simulated electron diffraction (ED) obtained from the X-ray single-crystal data of **MOF-NiBr₂** (CCDC 2255298) with the data shown in (g), viewing along a [-101] direction. The calculated patterns (d, h) are identical except for an in-plane rotation and a minor tilt out of the exact viewing direction. $NiCl_2 \cdot 6H_2O$ to **CSB** molar ratio = 2:1 with **CSB** = 0.6 mM.

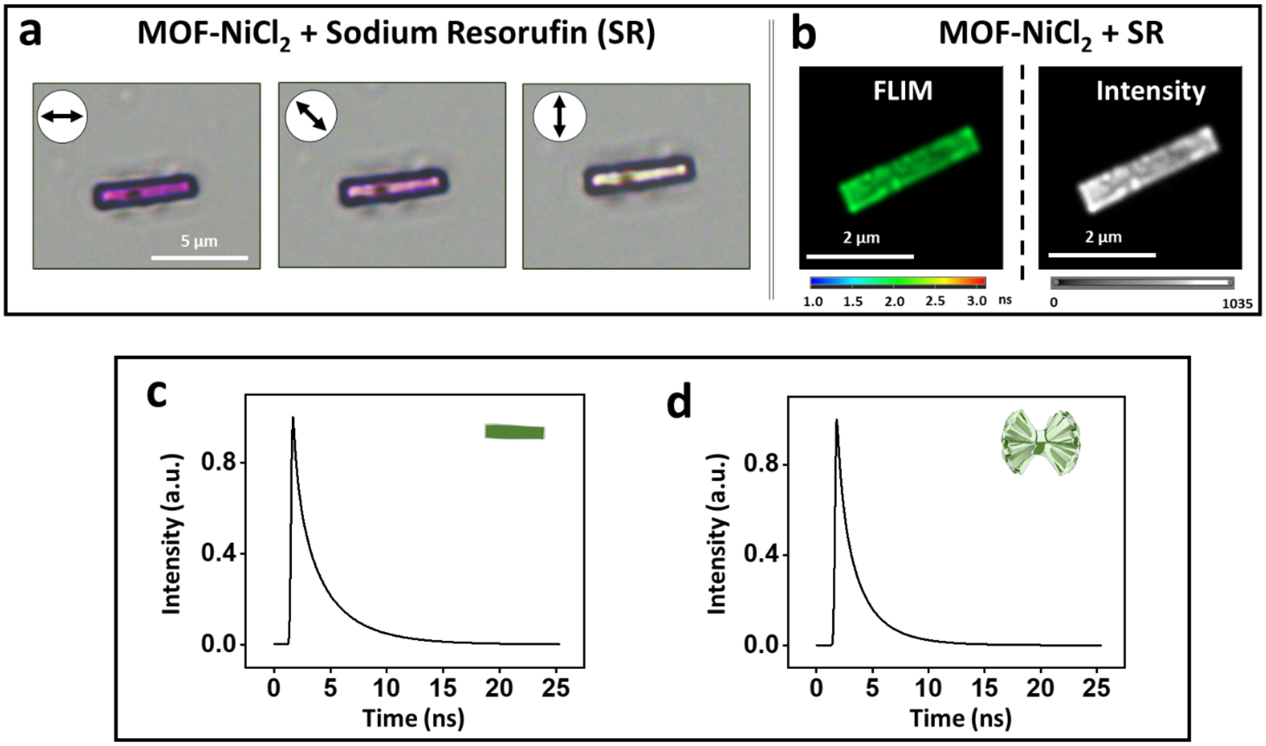

Figure S9. (a) Optical microscopy images of the of rod-like **MOF-NiCl₂** crystals (minor product; **Figure S2c,d**) functionalized with sodium resorufin (**SR**). (b) Confocal fluorescence lifetime imaging microscopy (FLIM) data (left) and fluorescence intensity image of crystals functionalized with the sodium resorufin (right). (c and d) Confocal fluorescence lifetime imaging microscopy (FLIM) spectra of **SR** functionalized (c) rod-like and (d) sheaf-like **MOF-NiCl₂** (λ_{ex} = 570 nm, λ_{em} = 650–700 nm). These crystals were obtained by immersing in an ethanol solution of **SR** (8.9×10^{-5} M) for 1 h. The $NiCl_2 \cdot 6H_2O$ to **CSB** molar ratio = 2:1, with **CSB** = 0.6 mM.

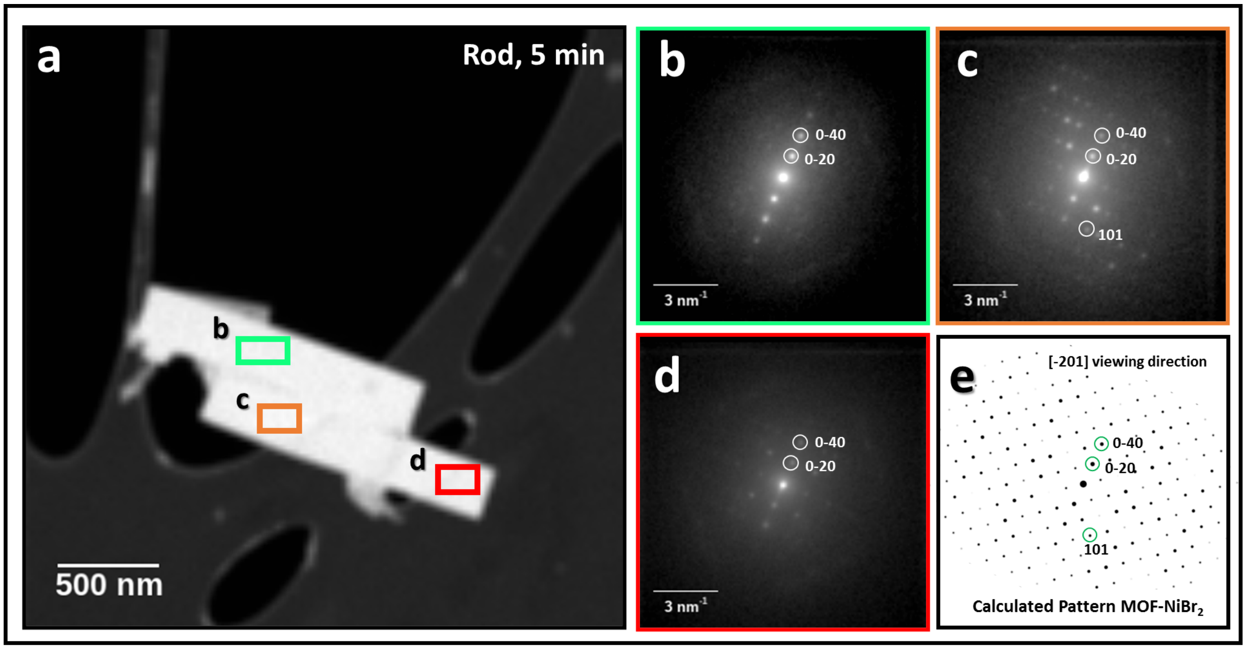

Figure S10. Scanning nanobeam electron diffraction (NBED) of **MOF-NiCl₂** formed after 5 min under solvothermal reaction conditions (105 ºC): $NiCl_2{\cdot}6H_2O$ to **CSB** molar ratio = 2:1, with **CSB** = 0.6 mM. (b-d) Sum diffraction patterns for areas marked in (a) reveal the single crystal nature of the rods with identical crystallographic habit planes across different nanorods. (e) Matching simulated electron diffraction (ED) pattern obtained from the X-ray single-crystal data of **MOF-NiBr₂** (CCDC 2255298) with the data shown in (c).

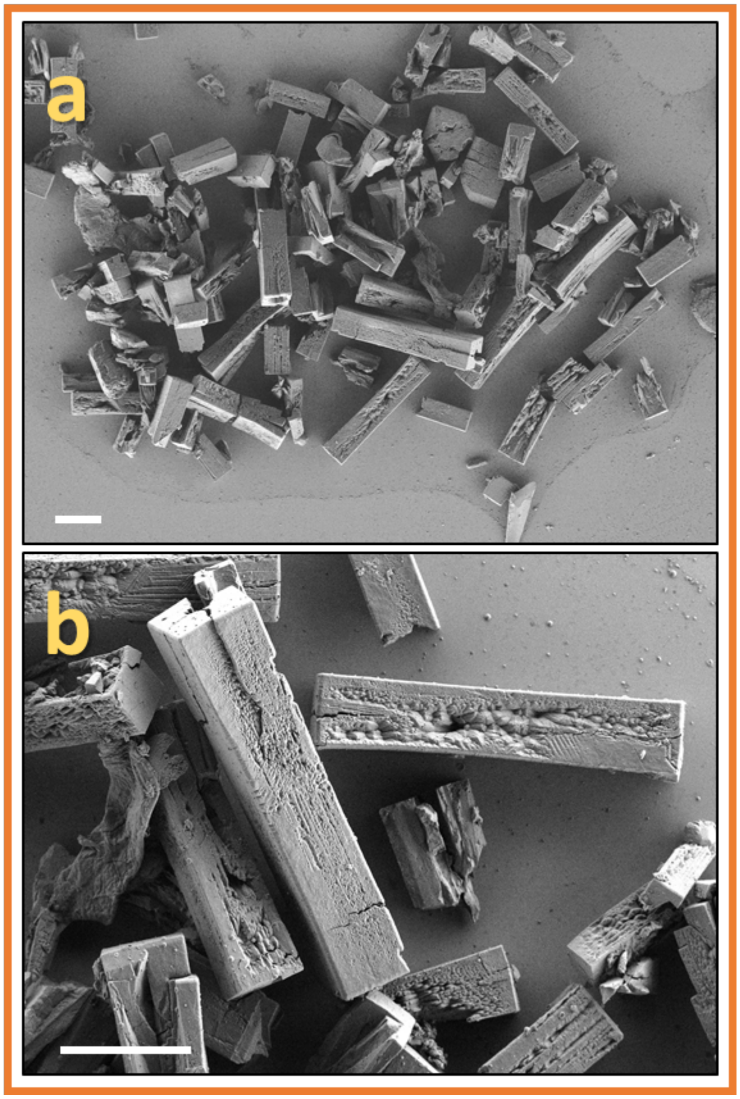

Figure S11. (a,b) Scanning electron microscopy (SEM) images of **MOF-NiCl₂**. The $NiCl_2 \cdot 6H_2O$ to **CSB** molar ratio = 0.5:1, with **CSB** = 0.6 mM. Scale bar = 20 μm.

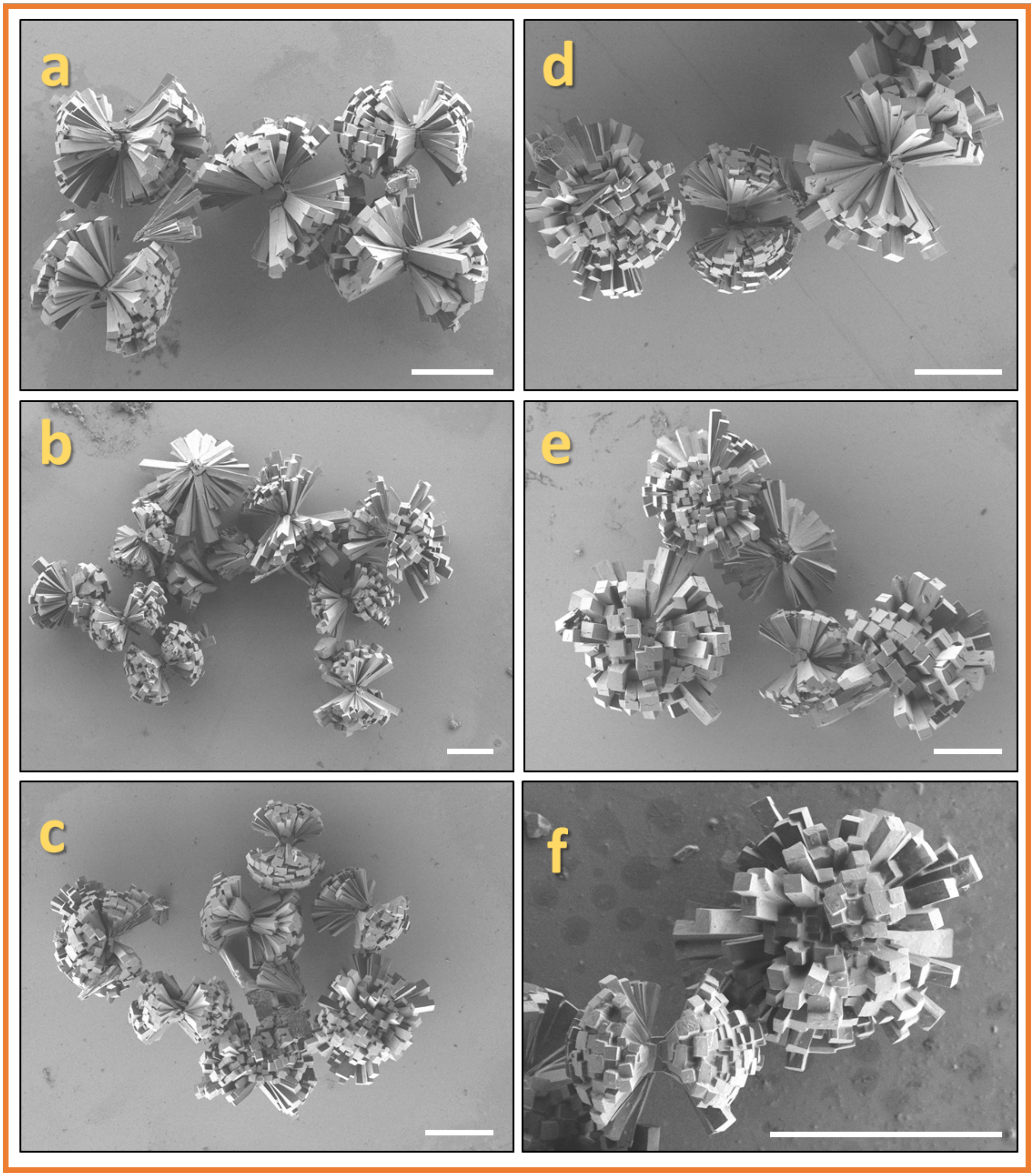

Figure S12. (a-f) Representative scanning electron microscopy (SEM) images of **MOF-NiCl$_2$**. The $NiCl_2 \cdot 6H_2O$ to **CSB** ratio = 1:1, with **CSB** = 0.6 mM. Scale bar = 20 μm.

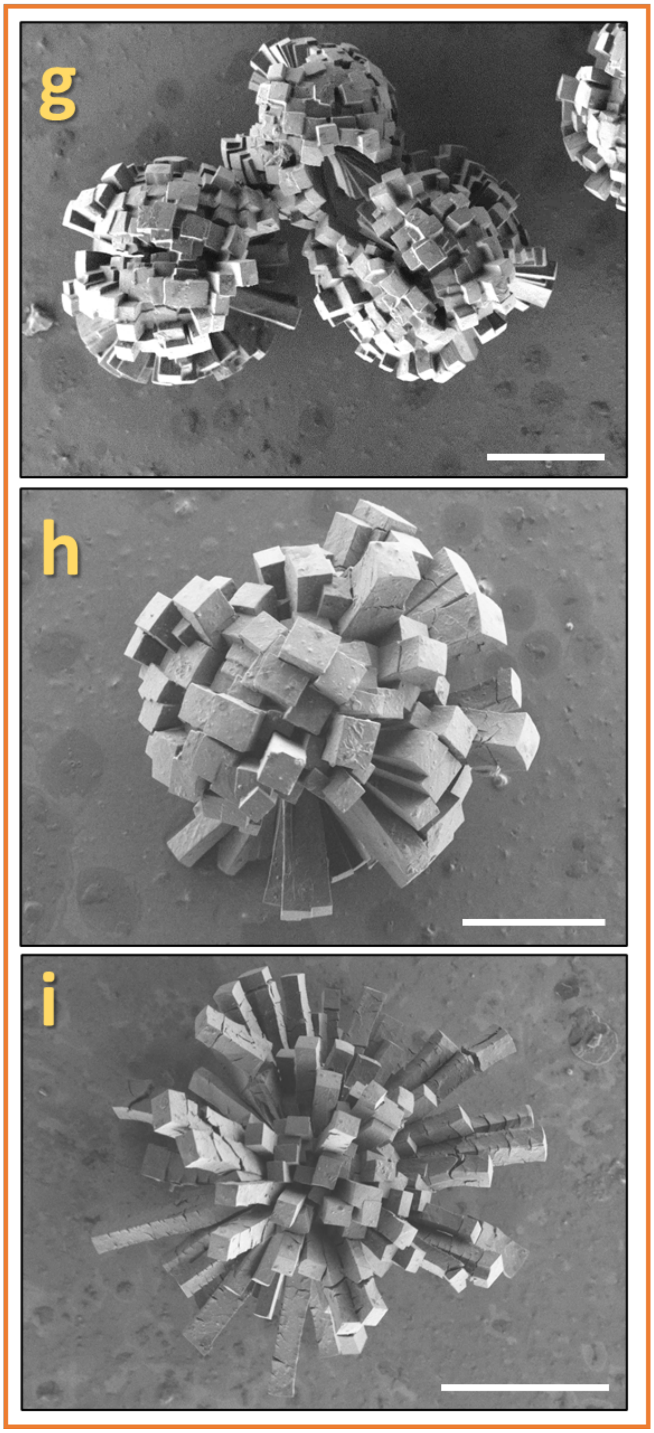

Figure S12 (Cont.). (g-i) Scanning electron microscopy (SEM) images of **MOF-NiCl$_2$** (spherulite). The $NiCl_2 \cdot 6H_2O$ to **CSB** molar ratio = 1:1, with **CSB** = 0.6 mM. Scale bar = 20 μm.

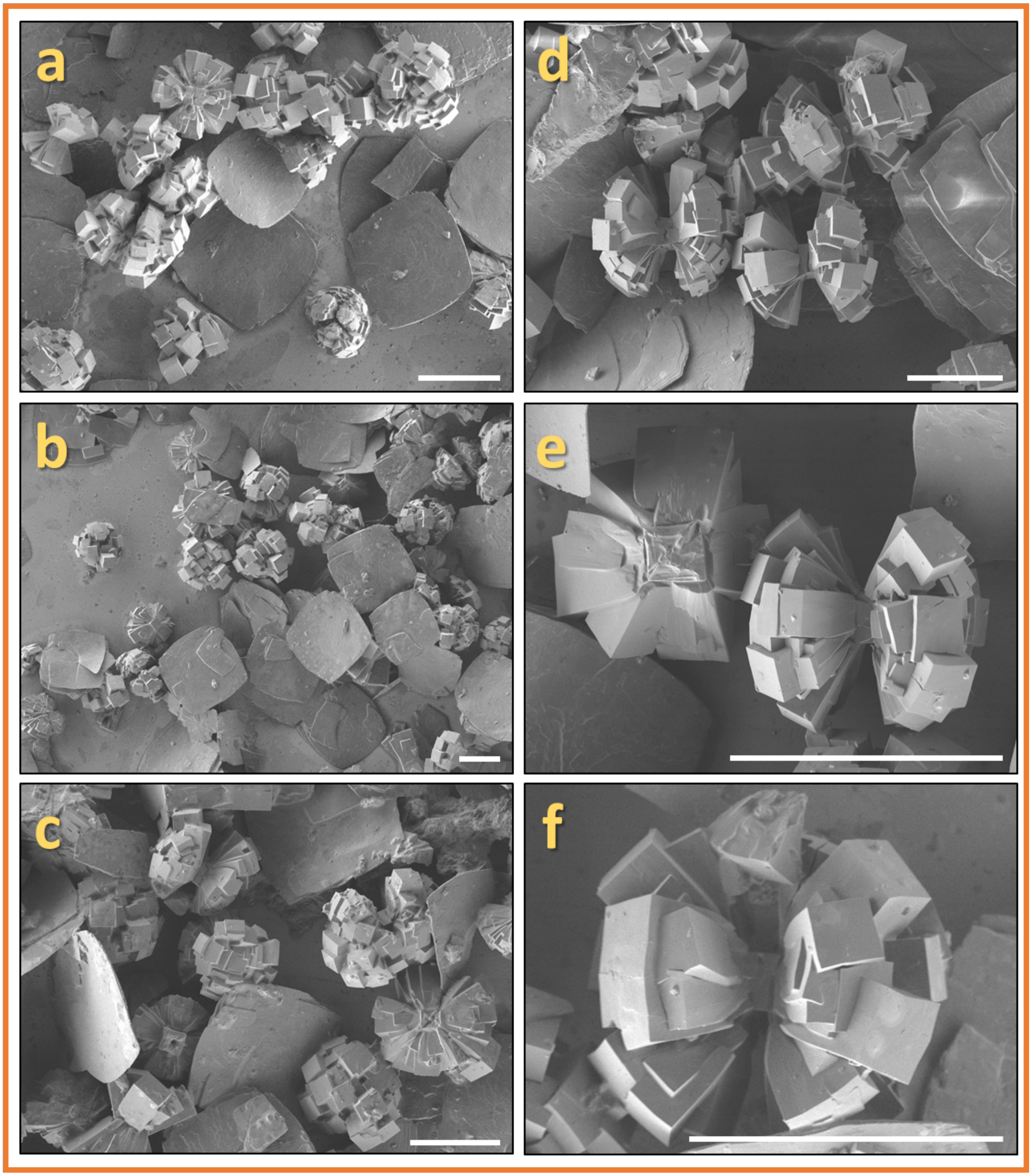

Figure S13. (a-f) Scanning electron microscopy (SEM) images of **MOF-NiCl$_2$**. The $NiCl_2 \cdot 6H_2O$ to **CSB** molar ratio = 4:1, with **CSB** = 0.6 mM. Scale bar = 20 μm.

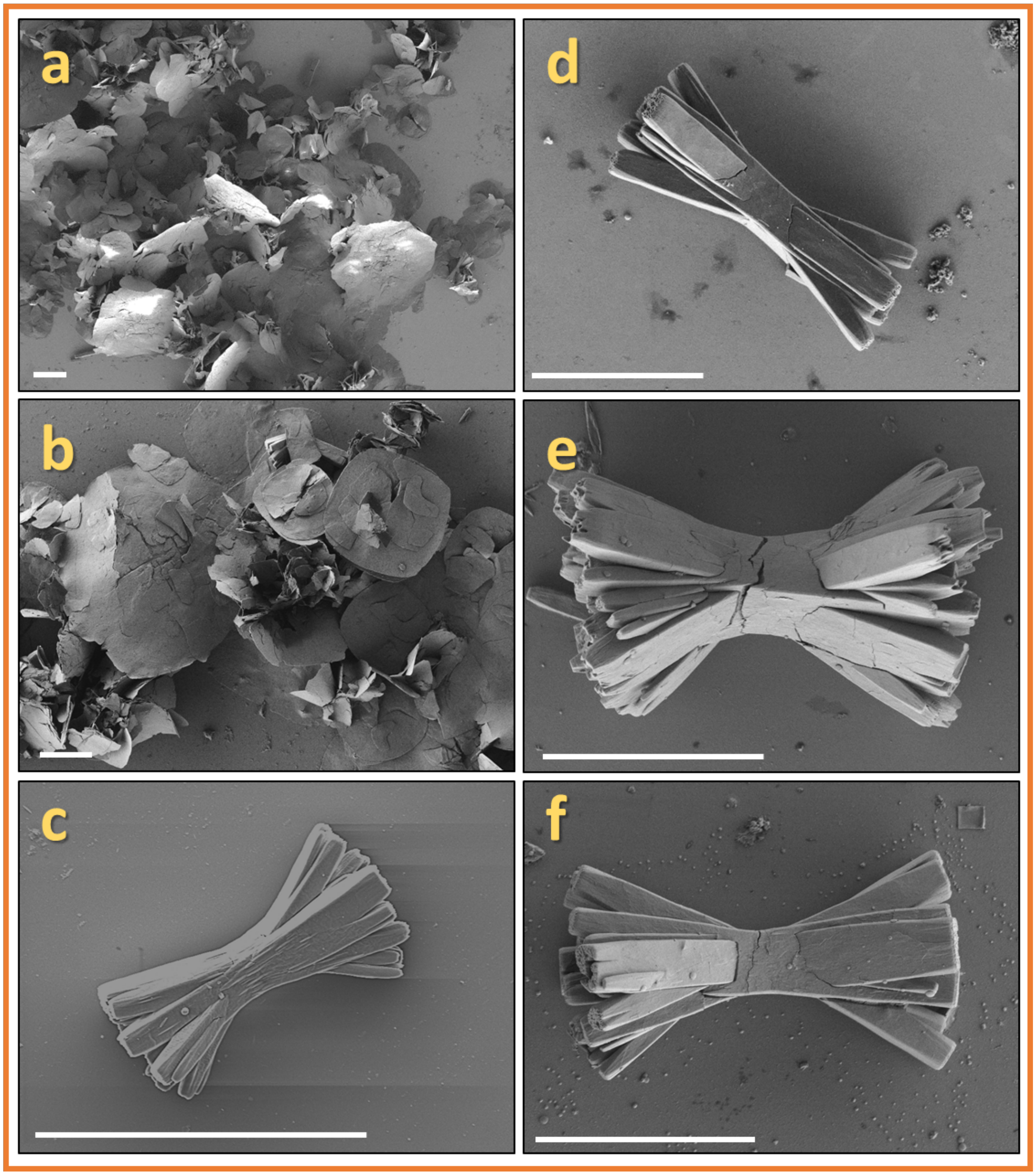

Figure S14. (a-f) Scanning electron microscopy (SEM) images of **MOF-NiCl$_2$**. The $NiCl_2 \cdot 6H_2O$ to **CSB** molar ratio = 2:1, with **CSB** = 1.2 mM. Scale bar = 10 μm.

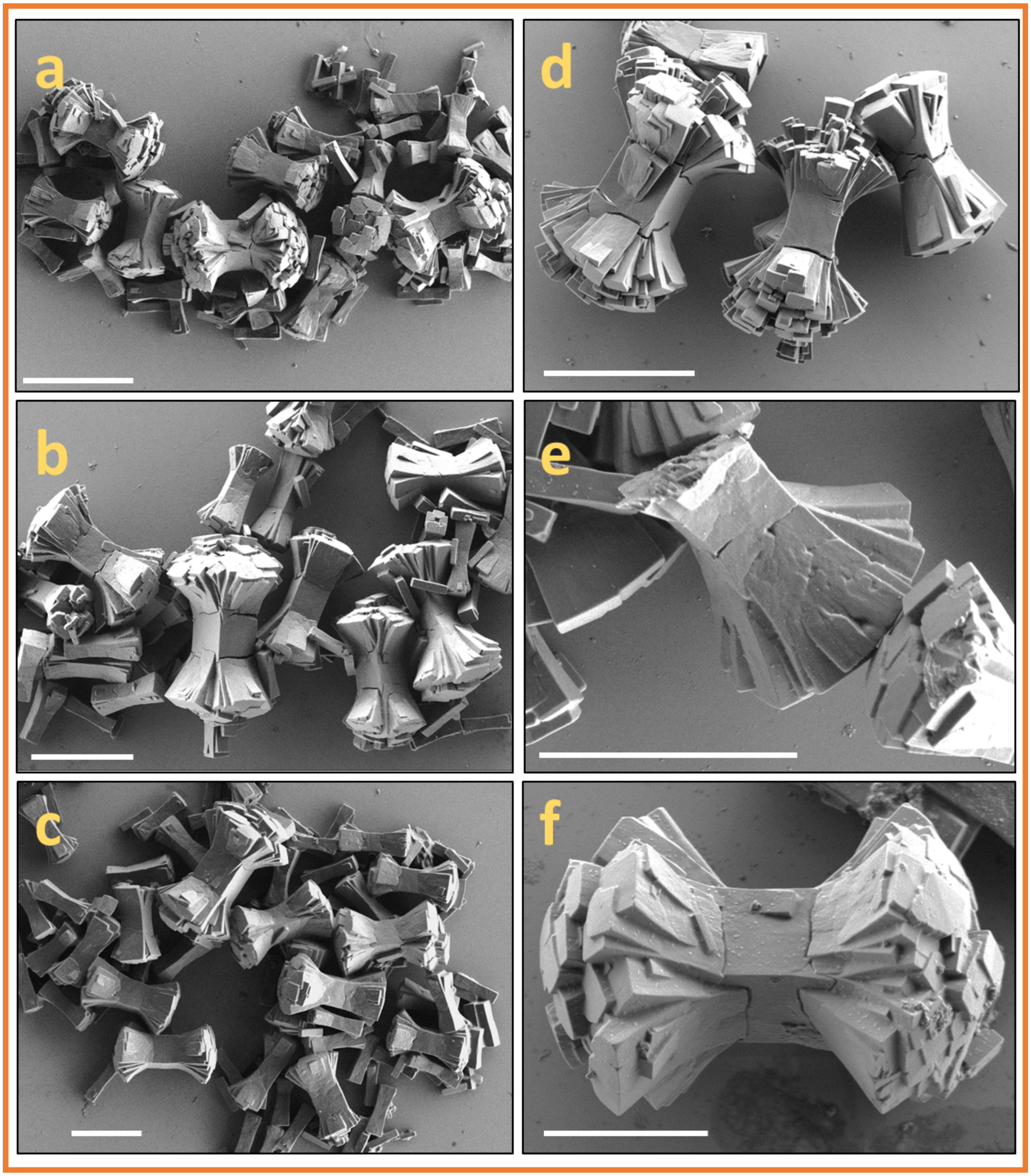

Figure S15. (a-f) Scanning electron microscopy (SEM) images of **MOF-NiCl$_2$**. The NiCl$_2$·6H$_2$O to **CSB** molar ratio = 2:1, with **CSB** = 0.81 mM. Scale bar = 10 μm.

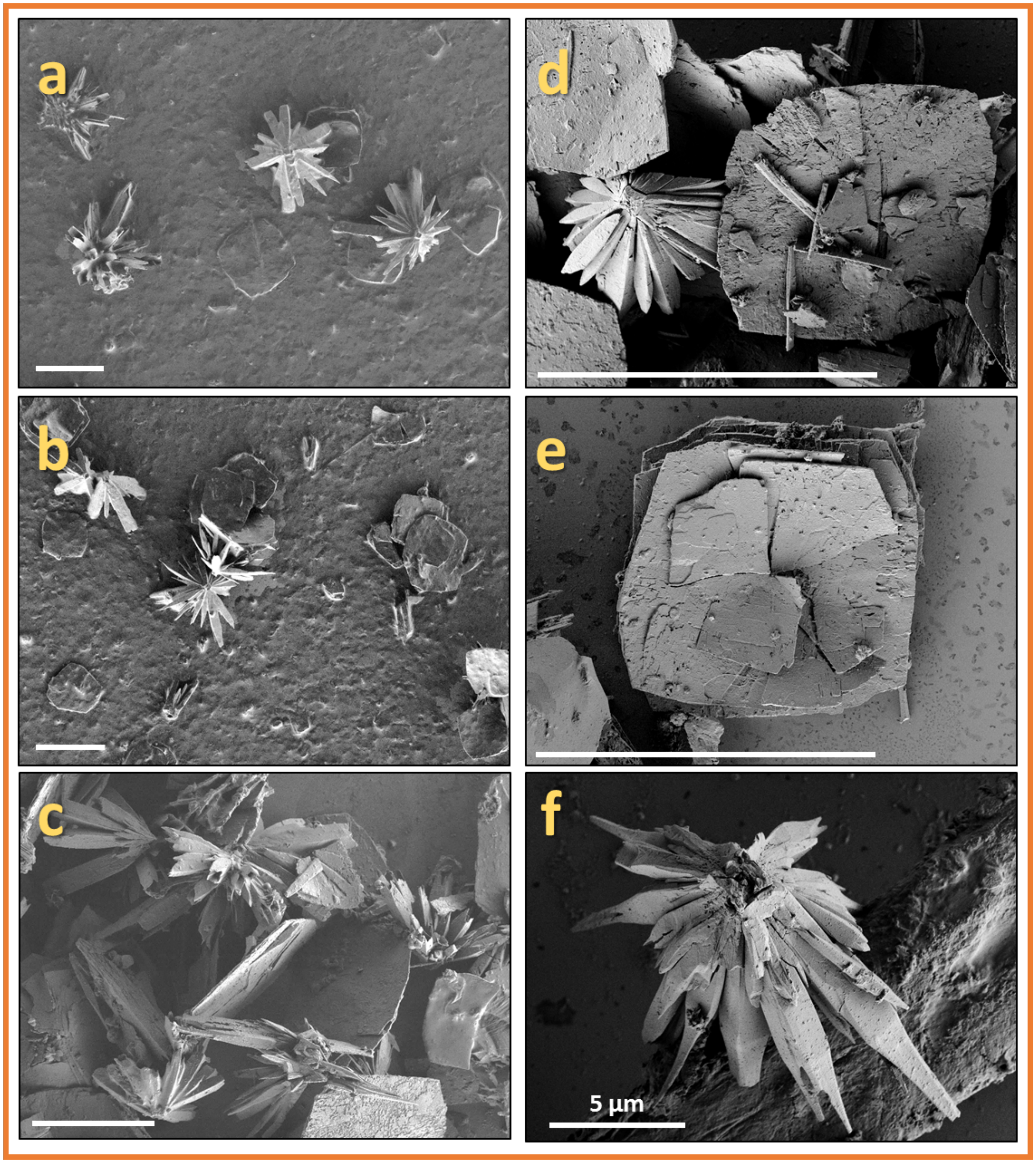

Figure S16. (a-f) Scanning electron microscopy (SEM) images of **MOF-NiCl$_2$**. The $NiCl_2 \cdot 6H_2O$ to **CSB** molar ratio = 2:1, with **CBS** = 0.42 mM. Scale bar = 15 μm (a-e).

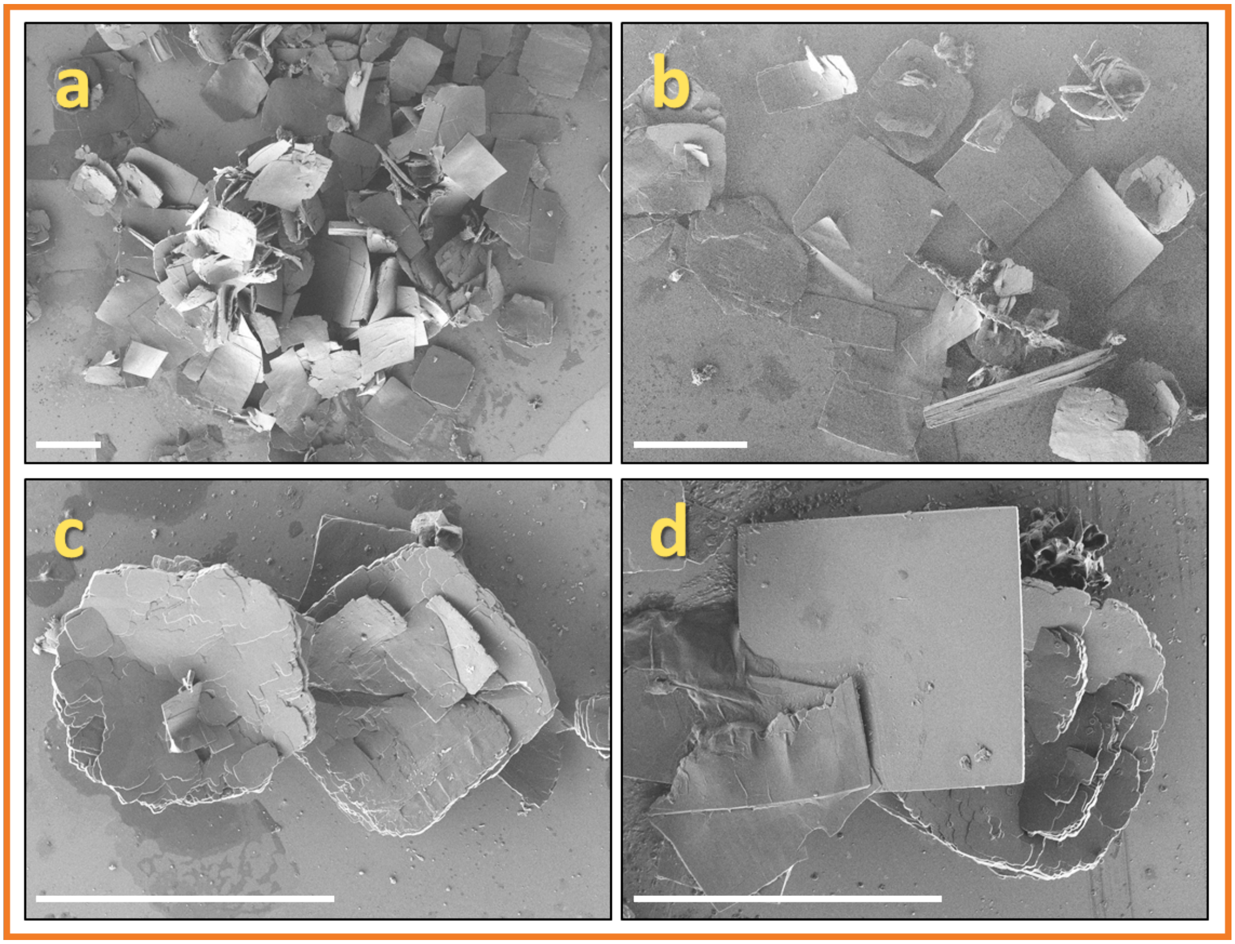

Figure S17. (a-d) Scanning electron microscopy (SEM) images of **MOF-NiCl$_2$**. The $NiCl_2 \cdot 6H_2O$ to **CSB** molar ratio = 2:1, with **CSB** = 0.15 mM. Scale bar = 50 μm.

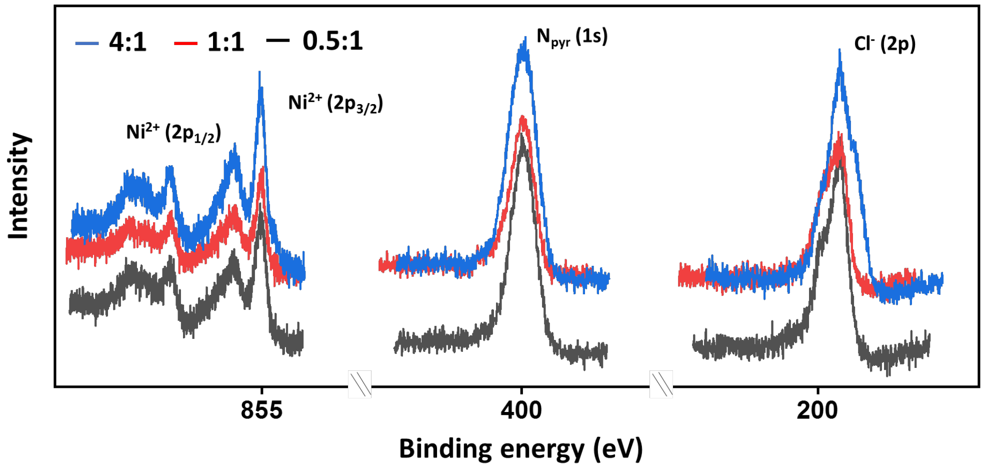

Figure S18. X-ray photoelectron spectroscopy (XPS) spectra of **MOF-NiCl$_2$** formed using three different $NiCl_2 \cdot 6H_2O$ to **CSB** molar ratios. Blue trace: $NiCl_2 \cdot 6H_2O$ to **CSB** molar ratio = 4:1. Red trace: $NiCl_2 \cdot 6H_2O$ to **CSB** molar ratio = 1:1. Black trace: $NiCl_2 \cdot 6H_2O$ to **CSB** molar ratio = 0.5:1, with **CSB** = 0.6 mM.

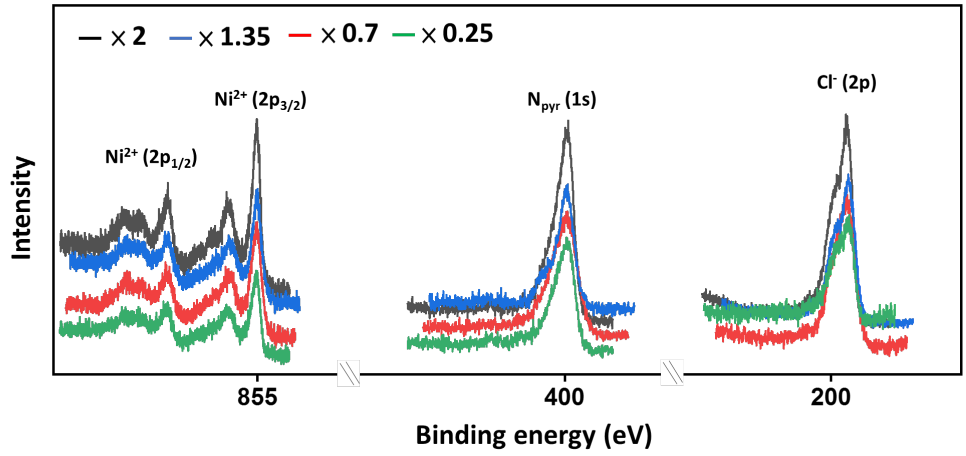

Figure S19. X-ray photoelectron spectroscopy (XPS) spectra of **MOF-NiCl$_2$** formed using four different concentrations of **CSB**. The molar ratio of $NiCl_2 \cdot 6H_2O$ to **CSB** was kept at 2:1. The concentrations were varied as follows: black trace: **CSB** = 1.2 mM (× 2), blue trace: **CSB** = 0.81 mM (× 1.35), red trace: **CSB** = 0.42 mM (× 0.7), green trace: **CSB** = 0.15 mM (× 0.25).

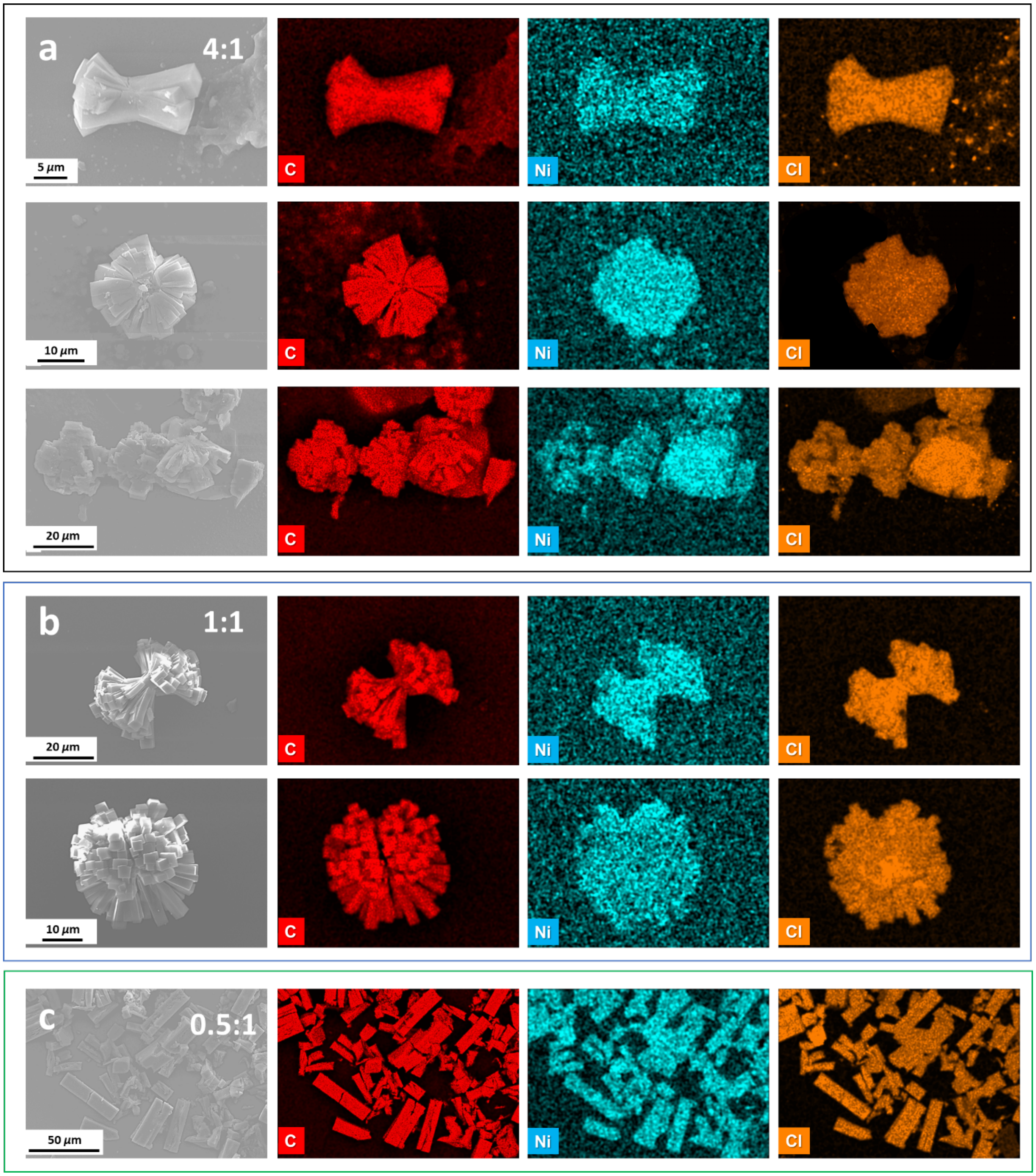

Figure S20. (a-c) Scanning electron microscopy (SEM) images and energy-dispersive X-ray spectroscopy (EDS) intensity maps of **MOF-NiCl₂** prepared using three different $NiCl_2 \cdot 6H_2O$ to **CSB** molar ratios: (a) $NiCl_2 \cdot 6H_2O$:**CSB** = 4:1. (b) $NiCl_2 \cdot 6H_2O$:**CSB** = 1:1. (c) $NiCl_2 \cdot 6H_2O$: **CSB** = 0.5:1, with **CSB** = 0.6 mM.

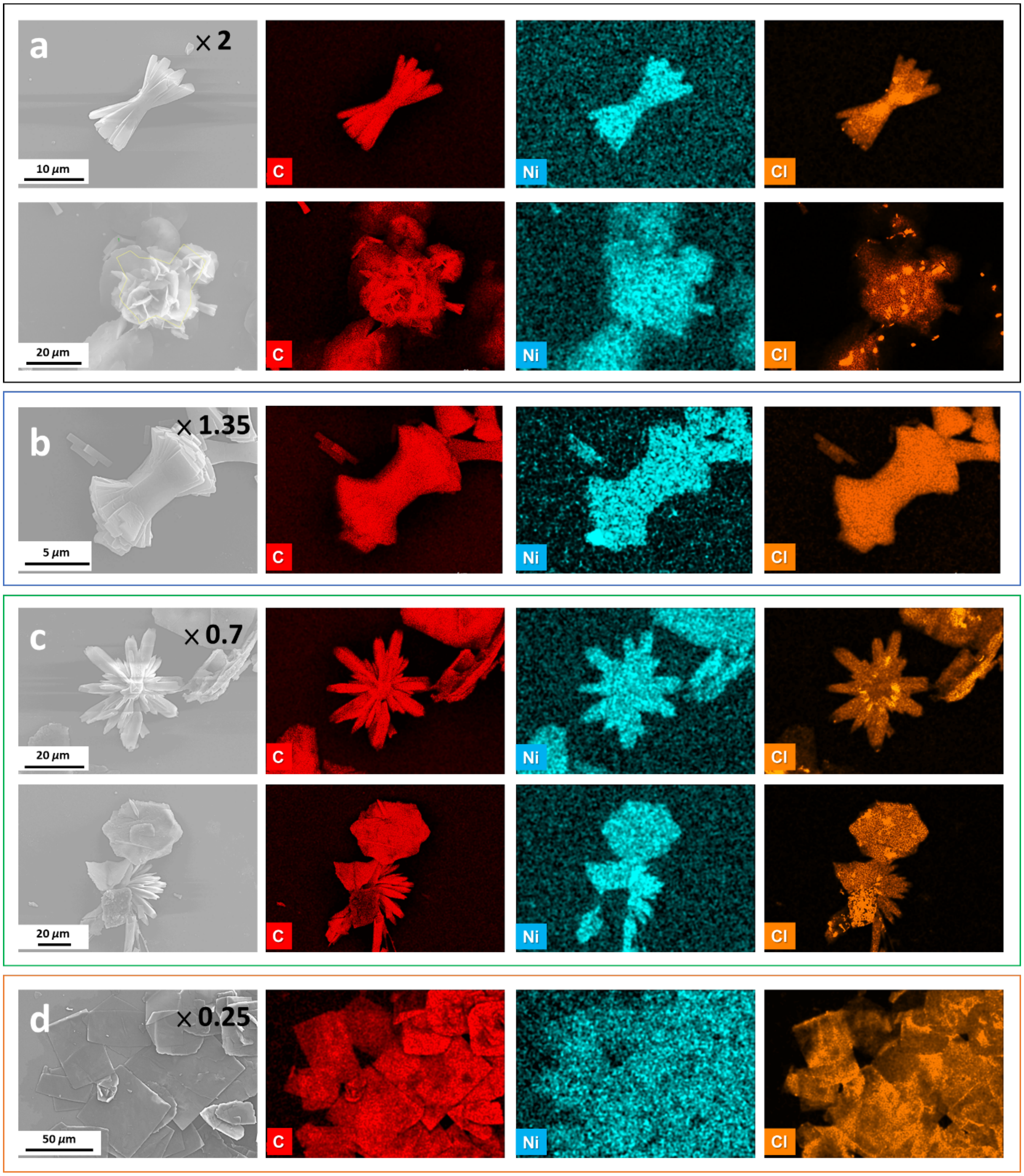

Figure S21. (a-d) Scanning electron microscopy (SEM) images and energy-dispersive X-ray spectroscopy (EDS) intensity maps of **MOF-NiCl$_2$**. The molar ratio of $NiCl_2 \cdot 6H_2O$ to **CSB** was kept at 2:1 and the amounts were varied as follows: (a) **CSB** = 1.2 mM, (b) **CSB** = 0.81 mM, (c) **CSB** = 0.42 mM, (d) **CSB** = 0.15 mM.

References

[S1] O. Chovnik, S. R. Cohen, I. Pinkas, L. Houben, T. E. Gorelik, Y. Feldman, L. J. W. Shimon, M. A. Iron, M. Lahav, M. E. van der Boom, *ACS Nano* **2021**, *15*, 14643–14652.

[S2] G. Beamson, D. Briggs, High Resolution XPS of Organic Polymers, the Scienta ESCA 300 Database, John Wiley and Sons; **1992**.

[S3] J. F. Moulder, W. F. Stickle, P. E. Sobol, K. D. Bomben, Handbook of X-ray Photoelectron Spectroscopy, Perkin-Elmer Corporation; **1992**.

[S4] G. M. Sheldrick, Crystal Structure Refinement with SHELXL. *Acta Cryst.* **2015**, *C71*, 3–8.

[S5] G. M. Sheldrick, SHELXT - Integrated Space-group and Crystal-structure Determination, Acta Cryst. **2015**, *A71*, 3-8.

[S6] G. M. Sheldrick, SHELXT-2013, Program for the Solution of Crystal Structures; University of Göttingen, Göttingen, Germany **2013**.
